# Effects of MRI-Navigated Intermittent Theta Burst Stimulation on Task Performance, Subjective Workload, and EEG Responses During Prolonged Simulated Flight: A Randomized Crossover Study

**DOI:** 10.3390/bioengineering13090997

**Published:** 2026-08-27

**Authors:** Zilong Liu, Yue Gong, Jing Dai, Yang Liu, Yilong Qin, Lin Yang, Hang Wang, Yang Yang, Chunchen Wang, Xinsheng Cao

**Affiliations:** Department of Aerospace Medicine, Air Force Medical University, Xi’an 710032, China

**Keywords:** intermittent theta burst stimulation, dorsolateral prefrontal cortex, simulated flight, NASA-TLX, EEG spectral power, sustained performance

## Abstract

Prolonged simulated flight is accompanied by progressive performance decay and escalating subjective workload. Whether intermittent theta burst stimulation (iTBS) over the left dorsolateral prefrontal cortex (DLPFC) can sustain performance and attenuate workload during sustained human–machine interaction remains unclear. To address this gap, we conducted a randomized, order-balanced, within-subject crossover study in which 25 healthy men completed a 60-min simulated flight (three 20-min phases) under MRI-navigated active iTBS (600 pulses, 80% of the resting motor threshold), sham iTBS, or no stimulation, with concurrent electroencephalography (EEG) recording. Objective performance was quantified using a composite performance score (CPS), and subjective workload was indexed by the NASA Task Load Index (NASA-TLX). Behaviorally, significant condition × phase interactions showed that active iTBS yielded higher CPS than both comparators in Phases 2–3 and lower NASA-TLX scores in Phase 3. At the neural level, task-state EEG revealed relative reductions in frontocentral theta and fronto-parieto-occipital alpha power under active iTBS in the later phases. Exploratory analyses further suggested that greater frontal alpha reductions tended to accompany larger CPS gains, although this association did not survive FDR correction. These findings suggest that MRI-navigated left-DLPFC iTBS was associated with better maintenance of task performance and lower subjective workload during the later stages of a prolonged simulated flight task. Given the small, homogeneous sample and the absence of real-flight scenarios, these results should be interpreted as preliminary laboratory evidence rather than evidence for operational efficacy or direct neuroergonomic application.

## 1. Introduction

Continuous control tasks during the cruise phase—such as maintaining heading, altitude, and airspeed—require operators to sustain stable output for tens of minutes or even hours in a monotonous, high-fidelity environment with delayed feedback. From the perspective of aviation neuroergonomics, such tasks not only on sensorimotor integration but also impose progressively increasing demands on sustained attention, executive control, and spatial working memory [1,2]. As time on task accumulates, operators typically exhibit a characteristic performance decay curve—prolonged reaction times, increased errors, and coarser control laws—accompanied by a steep rise in subjective workload [3]. Traditional countermeasures, such as scheduled rest breaks, automation take-over, or anticipatory skill training, can alleviate fatigue to some extent; however, they act mainly at the level of environmental engineering or task management, and it remains untested whether prefrontal neuromodulation is associated with changes in behavioral performance, subjective workload, or EEG activity during prolonged monotonous operations. Whether modulating prefrontal executive networks can influence task-state evolution during sustained simulated control therefore remains an open empirical question at the intersection of engineering psychology and neuromodulation.

Mechanistically, the mental fatigue induced by prolonged continuous operation is not a simple matter of “energy exhaustion”; rather, it involves a multisystem reorganization that includes diminished top-down regulatory efficiency of the prefrontal cortex (PFC), hyperactivation of the salience network, and aberrant intrusion of the default mode network (DMN) [4,5]. Specifically, the dorsolateral prefrontal cortex (DLPFC), as a core node of the frontoparietal control network, plays a hub role in maintaining goal-directed behavior, conflict monitoring, and working memory updating [6]. When DLPFC function declines with accumulating time on task, the brain–machine system often exhibits compensatory neural recruitment—that is, achieving the same behavioral output requires more widespread cortical activation or greater subjective effort [7]. This raises the possibility that non-invasive modulation of the DLPFC before task engagement may influence subsequent sustained-control states during prolonged tasks. However, whether such modulation translates into measurable behavioral or EEG differences under controlled simulated-task conditions remains an empirical question.

Intermittent theta burst stimulation (iTBS), a patterned form of repetitive transcranial magnetic stimulation designed to induce long-term potentiation (LTP)-like plasticity, offers a unique time-efficiency advantage: it delivers 600 pulses—50-Hz triplets repeated at 5 Hz—within approximately 3 min, transiently modulating cortical excitability in the target region. The left DLPFC, owing to its causal role in goal maintenance, distractor suppression, and cognitive flexibility, has become the most frequently adopted iTBS target in cognitive enhancement research [6,7]. Studies have shown that single or short-term iTBS can improve working memory updating, task switching, and inhibitory control in discrete psychological tests [8]. However, a notable methodological gap exists in the current literature: the vast majority of iTBS–cognition studies employ minute-long, trial-based tasks (e.g., N-back, Stroop, and Go/No-Go), whose task structure is dominated by discrete stimulus–response events and lacks the real-time sensorimotor integration and prolonged monotonous load required for continuous closed-loop tracking. Whether the results of these short-duration tasks can be extrapolated to continuous human–machine interaction scenarios lasting more than 1 h remains unknown.

In the field of flight simulation, which more closely resembles the present paradigm, a recent study applying high-definition transcranial direct current stimulation (HD-tDCS) over the bilateral DLPFC found that anodal stimulation improved flight performance and cognitive flexibility [9]. That study provided an important proof of concept for the application of prefrontal non-invasive modulation in aviation ergonomics; however, HD-tDCS and iTBS differ fundamentally in their neurophysiological mechanisms: the former relies on sustained subthreshold electric-field polarization to alter cortical resting membrane potentials, with a relatively broad temporal window of effect, whereas the latter depends on spike-timing-dependent plasticity, inducing long-term changes in synaptic efficacy by precisely mimicking endogenous theta oscillatory rhythms [10,11]. More importantly, the two techniques differ in their dynamics—the subthreshold polarization of tDCS acts mainly during stimulation and its after-effects are relatively nonspecific, whereas iTBS induces spike-timing-dependent synaptic plasticity with a more complex after-effect time course that can interact with subsequent neural activity states. Therefore, the positive results of HD-tDCS in short-duration flight tasks cannot be readily transferred to the combination of iTBS and prolonged flight.

The behavioral effects of iTBS may depend on the ongoing brain and task state. State-dependent accounts of non-invasive brain stimulation suggest that the direction and magnitude of post-stimulation changes can vary according to the activation state of the targeted network before and during subsequent task engagement [7,12]. From this perspective, the behavioral consequences of DLPFC iTBS may be more likely to emerge when sustained task demands accumulate rather than immediately at task onset [13]. Accordingly, we hypothesized that active iTBS would be associated with differences in behavioral performance, subjective workload, and EEG activity during the middle and late phases of a prolonged simulated flight task. This hypothesis was framed as an empirical prediction about task-state evolution, not as a direct test of synaptic plasticity or neural-efficiency mechanisms. To date, however, no study has systematically tracked the temporal evolution of iTBS behavioral effects during a continuous control task lasting more than 1 h, nor has any study linked such effects to concurrent neurophysiological evidence.

To elucidate the physiological mechanisms of iTBS during prolonged operations, electrophysiological measures capable of mapping cortical dynamics in real time must be introduced. With its millisecond-level temporal resolution, electroencephalography (EEG) has become the tool of choice for monitoring operator state in neuroergonomics [14]. In research on sustained attention and cognitive load, frontocentral theta (4–8 Hz) power is generally regarded as a sensitive index of cognitive control demands and compensatory effort—prefrontal theta activity typically increases markedly as fatigue accumulates or conflict rises—whereas parieto-occipital alpha (8–13 Hz) power exhibits a complex pattern during sustained vigilance tasks: alpha suppression during the early task-engagement period reflects active attentional investment, whereas the later alpha rebound is often regarded as a marker of increased cortical idling, attentional disengagement, or excessive DMN coupling [15,16]. Therefore, if relative reductions in late-phase frontocentral theta and parieto-occipital alpha power are observed under the iTBS condition, this would indicate behavioral benefit and provide electrophysiological evidence that the stimulation was associated with a different pattern of task-related neural dynamics, rather than a simple reduction in neural activation cost [17]. However, previous iTBS cognition studies have mostly focused on resting-state or extremely brief task-state EEG after stimulation, lacking dynamic spectral tracking under prolonged continuous operation.

In summary, existing research has gaps at least at three levels: (1) whether the benefits of iTBS can be extrapolated from minute-scale discrete tasks to hour-scale continuous control tasks; (2) how the behavioral effects of iTBS evolve over time during prolonged monotonous operations, and whether they follow the mid-to-late-phase advantage pattern predicted by the state-dependency hypothesis; and (3) whether iTBS-related EEG spectral dynamics can explain its neuroergonomic mechanism. To fill these gaps, this study established a randomized, order-balanced crossover experimental platform combining MRI-navigated iTBS with a 60-min continuous simulated flight task and concurrent EEG. By comparing three conditions—active stimulation, sham stimulation, and no stimulation—and by dividing the flight task into three 20-min phases, we proposed the following hypotheses: (1) active iTBS over the left DLPFC can significantly attenuate the time-accumulated deterioration of flight performance, and this effect emerges mainly in the middle and late phases of the operation; (2) the intervention can concurrently weaken the progressive rise in subjective workload, particularly at the end of the task; and (3) the above behavioral effects are accompanied by changes in task-state EEG spectral features, manifested as relative reductions in frontocentral theta and fronto-parieto-occipital alpha power in the middle and late phases, a pattern that would be consistent with a different mode of task-related neural dynamics under sustained cognitive demand.

## 2. Materials and Methods

### 2.1. Participants

A total of 25 healthy young male participants were recruited for this study. After screening, all participants met the experimental requirements and completed the full test battery, yielding a final sample of N = 25 for statistical analysis. An a priori sample-size calculation was conducted using G*Power 3.1 before participant recruitment. For the prespecified primary behavioral outcomes, namely the composite performance score (CPS) and the NASA Task Load Index (NASA-TLX), a within-subject repeated-measures ANOVA was assumed with a moderate effect size of f = 0.28, α = 0.05, statistical power of 1 − β = 0.80, and a correlation of r = 0.50 among repeated measurements; the minimum required sample size was calculated as N = 22. Thus, the final sample of N = 25 was considered adequate for detecting prespecified moderate effects in the primary behavioral analyses. Additionally, a sensitivity power analysis for paired post hoc *t*-tests indicated that N = 25 provided 80% power to detect a within-subject effect size of Cohen’s d_z_ ≥ 0.58 at a two-sided α = 0.05. The mean age was 23 ± 2.16 years, and the mean duration of education was 16 ± 2.26 years. The inclusion criteria were as follows: aged 20–30 years; right-handed; normal or corrected-to-normal vision; and able to understand the experimental instructions and complete the complex simulated flight task. The exclusion criteria were as follows: any contraindication to transcranial magnetic stimulation (e.g., a family history of epilepsy, intracranial metallic implants, or a cardiac pacemaker); use of medications affecting central nervous system excitability within the past 12 months; a history of severe traumatic brain injury; and the presence of mood disorders (anxiety or depression) or sleep disorders. Participants were required to abstain from alcohol, excessive caffeine intake, and strenuous exercise within 24 h before the experiment and to maintain a regular sleep–wake schedule. The study protocol was approved by the Ethics Committee of Xijing Hospital (approval No. KY20260190), and all participants signed written informed consent forms.

### 2.2. Experimental Design

**Figure 1:**
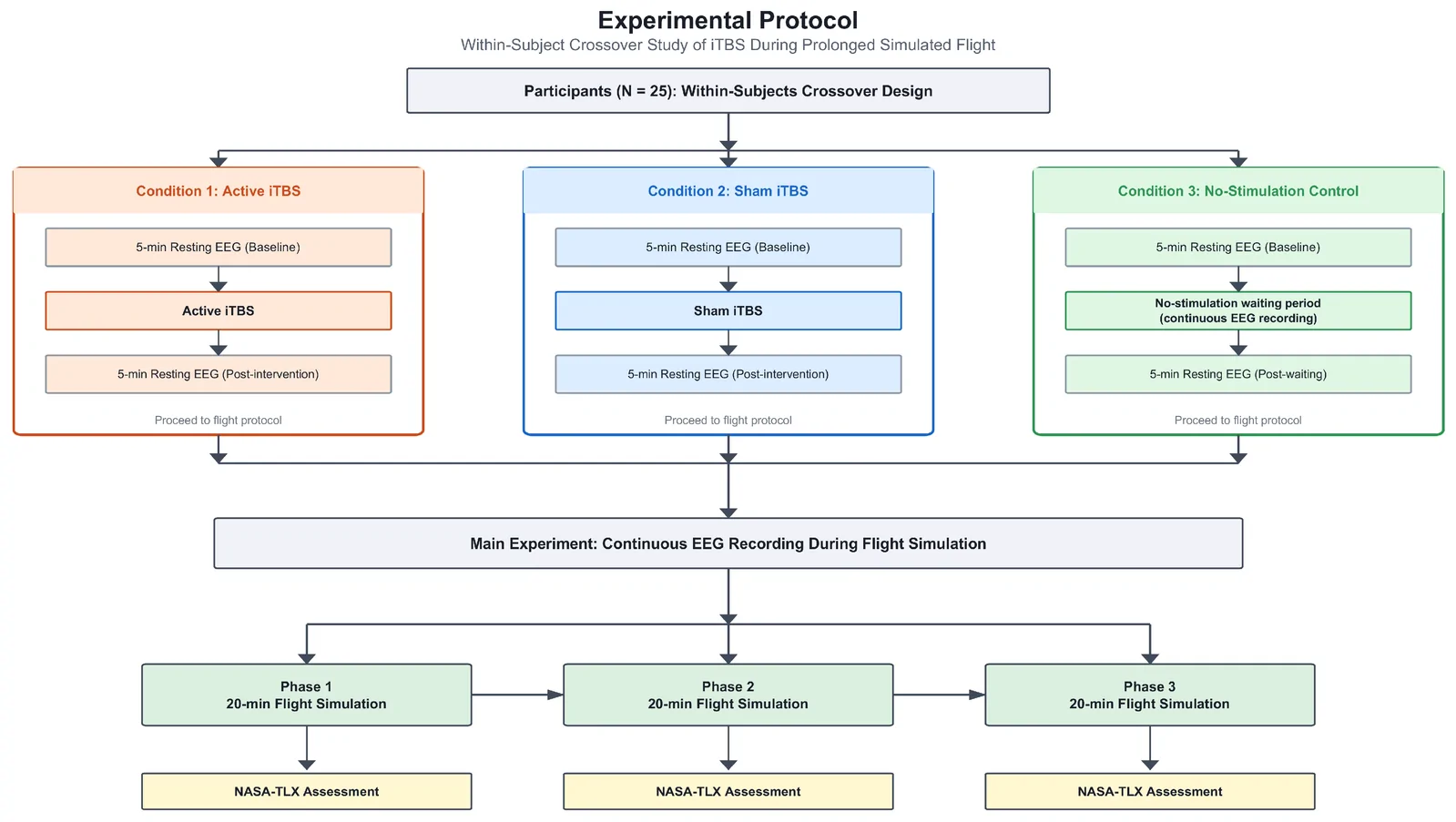
Experimental protocol. In each of the three conditions, participants first underwent a 5-min baseline resting-state EEG recording. This was followed by active iTBS, sham iTBS, or a no-stimulation waiting period, respectively, and then by a second 5-min resting-state EEG recording. Participants subsequently completed a 60-min continuous simulated flight task divided into three consecutive 20-min phases (Phases 1–3), during which task-state EEG was recorded continuously. Immediately after each flight phase, participants completed the NASA-TLX to assess subjective workload during that phase.

This study adopted a randomized, within-subject crossover design with a Latin-square arrangement intended to balance the position of each condition across study periods. Each participant completed the following three experimental sessions, each separated by a washout period of at least 7 days: (1) an active stimulation condition, in which individualized MRI-navigated iTBS intervention was administered; (2) a sham stimulation condition, in which a sham coil was used to mimic the timing, coil placement, and audible clicking cues of active iTBS while producing no intended cortical stimulation; and (3) a no-stimulation control condition, in which no TMS procedure was administered. The order of experimental sessions was counterbalanced via a computer-generated 3 × 3 Latin square. Participants (N = 25) were sequentially assigned to one of three treatment sequences, resulting in an allocation of *n* = 8, *n* = 8, and *n* = 9 participants per sequence respectively (detailed in Supplementary Table S1). Allocation concealment was maintained by an independent researcher not involved in data collection. The standardized experimental procedure is illustrated in Figure 1.

### 2.3. MRI-Navigated iTBS Intervention Protocol

The resting motor threshold (RMT) was determined before the iTBS intervention using single-pulse TMS over the hand area of the left primary motor cortex. The relaxed right abductor pollicis brevis was used as the target muscle. The motor hotspot was identified as the scalp position at which single-pulse stimulation elicited the most stable motor-evoked potentials (MEPs). RMT was defined as the minimum stimulator output that evoked MEPs with a peak-to-peak amplitude of at least 50 μV in at least 5 of 10 consecutive trials.

The left dorsolateral prefrontal cortex (DLPFC) stimulation target was localized with a Brainsight neuronavigation system. For each participant, the MNI152 standard-space coordinates (−42, 44, 30) were transformed into the participant’s native MRI space based on the individual high-resolution T1-weighted structural image [18,19]. During stimulation, real-time neuronavigation was used to maintain the coil position and orientation relative to the individualized target. The target-position tolerance was approximately 2 mm, and coil orientation was maintained as closely as possible to the planned orientation. Active iTBS was delivered using a Magstim Rapid2 stimulator with a 70-mm figure-of-eight coil at 80% of individual RMT. The coil handle was oriented approximately 45° posterolaterally relative to the midsagittal plane. The standard iTBS protocol consisted of bursts of three pulses at 50 Hz repeated at 5 Hz, with each stimulation train lasting 2 s followed by an 8-s inter-train interval, yielding 600 pulses over approximately 190 s [20]. The simulated-flight task began approximately 5 min after the end of stimulation.

In the sham iTBS condition, a dedicated sham coil was positioned over the same target region and used with the same procedural timing as active iTBS. The sham coil was intended to mimic the clicking sound, coil placement, and general procedural context of stimulation; however, it could not fully reproduce the scalp somatosensory sensations associated with active iTBS.

### 2.4. Simulated Flight Task and Performance Assessment

#### 2.4.1. Flight Scenario and Task Procedure

The experiment was conducted on a high-fidelity simulated flight platform modeled on the glass cockpit of a narrow-body jet airliner, consisting of high-resolution displays, a flight control stick, and a throttle quadrant. Before the formal experiment, participants received 3 h of standardized training covering instrument recognition and coordinated three-axis control. The qualification criterion was defined as a coefficient of variation below 10% for every control parameter during a continuous 10-min test, so as to minimize learning effects.

**Figure 2:**
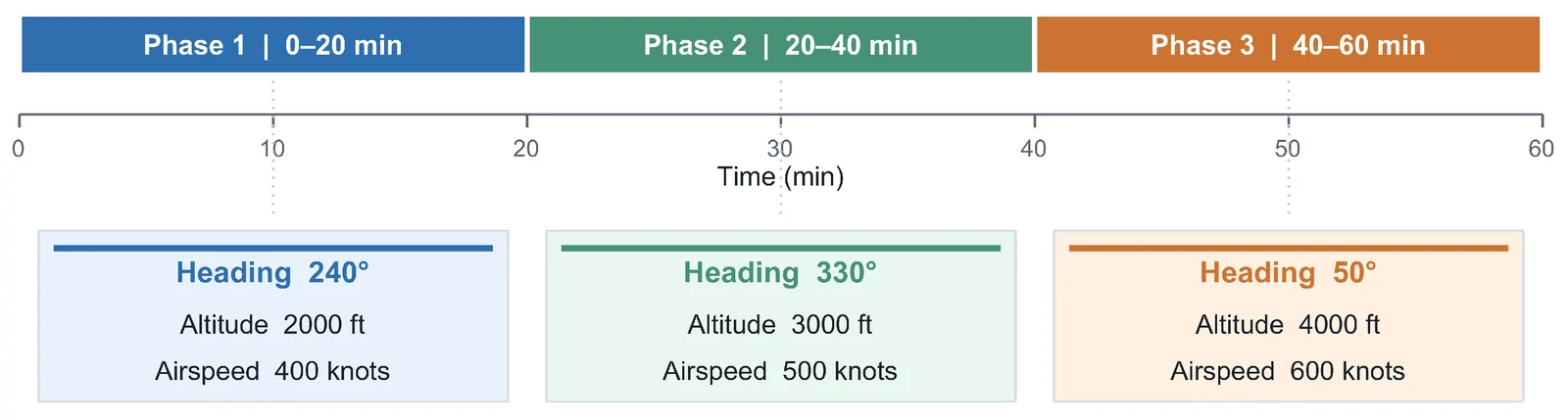
Target values of heading, altitude, and airspeed in each flight phase.

In the formal task, the total flight duration was 60 min, divided into three flight phases (Phases 1–3, 20 min each) (Figure 2). Participants were required to maintain preset cruise parameters: in Phase 1, heading 240°, altitude 2000 ft, and airspeed 400 knots; in Phase 2, heading 330°, altitude 3000 ft, and airspeed 500 knots; and in Phase 3, heading 50°, altitude 4000 ft, and airspeed 600 knots. The differences in target parameters among the three phases were intended to simulate the consecutive waypoint transitions of real cruise flight and to avoid overlearning resulting from maintaining a single parameter set; subsequent flight performance was compared using standardized scores computed on a common scale across all phases.

#### 2.4.2. Flight Performance Metrics

The flight task log recorded the real-time values of heading, altitude, and airspeed at a sampling rate of 10 Hz. The control errors of heading, altitude, and airspeed were computed separately for each participant, each condition, and each task phase. In the following equations, the subscript “actual” denotes the actual value of the corresponding variable, the subscript “target” denotes its target value, and N denotes the total number of samples.

Because heading is a circular variable, the heading error was computed in shortest-distance form:

(1) $$d_{heading}=\left|\left(\left(\theta_{actual}-\theta_{target}+180\right)\;mod\;360\right)-180\right|$$

The root mean square error (RMSE) of heading was then computed as follows:

(2) $${\mathrm{RMSE}}_{\mathrm{heading}}=\sqrt{\frac{1}{N}\sum_{i=1}^{N}d_{\mathrm{heading},i}^{2}}$$

The RMSEs of altitude and airspeed were computed in the same manner:

(3) $${RMSE}_{altitude}=\sqrt{\frac{1}{N}\sum_{i=1}^{N}{\left(h_{actual,i}-h_{target,i}\right)}^{2}}$$

(4) $${RMSE}_{airspeed}=\sqrt{\frac{1}{N}\sum_{i=1}^{N}{\left(v_{actual,i}-v_{target,i}\right)}^{2}}$$

For each participant, intervention condition, and flight phase, the heading, altitude, and airspeed RMSEs were standardized separately using pooled reference values calculated for the corresponding metric across all participant condition phase observations. Specifically, for each metric, the pooled reference distribution comprised 225 RMSE observations: 25 participants × 3 intervention conditions × 3 flight phases. This global standardization placed the three error metrics, which had different physical units, on a common within-study scale while preserving their between-condition and between-phase contrasts within the present dataset.

(5) $$Z_{k}=\frac{{RMSE}_{k}-\mu_{k}}{\sigma_{k}}$$

where *k* denotes heading, altitude, or airspeed; *μ_k_* and *σ_k_* denote the mean and standard deviation, respectively, of the corresponding RMSE across all 225 observations; and *Z_k_* denotes the standardized RMSE for a given participant, intervention condition, and flight phase. The three standardized error metrics were assigned equal weights and summed to form the composite error:

(6) $$CompositeError=Z_{heading}+Z_{altitude}+Z_{airspeed}$$

The equal-weight sum was selected as a transparent and reproducible prespecified approach in the absence of an externally validated weighting scheme, expert-derived weights, or an independent criterion for assigning different importance to heading, altitude, and airspeed errors. It should not be interpreted as an externally validated optimal weighting model. For ease of interpretation, the sign of the composite error was reversed so that a higher composite flight control performance score indicates better performance:

(7) $$P_{composite}=-CompositeError$$

Because the standardization parameters were estimated from the pooled observations in the present study, CPS is a within-study composite metric and its absolute values are sample-dependent. This approach was used to construct a common scale for the current analyses and does not constitute external validation of the composite score or its equal-weight specification.

### 2.5. NASA-TLX Workload Assessment

The NASA-TLX scale was used to quantitatively assess participants’ subjective workload during each flight phase. The scale provides an integrated evaluation of six dimensions: mental demand, physical demand, temporal demand, performance, effort, and frustration. Immediately after each 20-min flight phase, participants completed the NASA-TLX assessment using a tablet-based form. They first rated each dimension on a continuous 0–100 scale and then completed 15 pairwise comparisons of the relative importance of the six dimensions according to the standard NASA-TLX pairwise weighting procedure [21]. Each NASA-TLX assessment took approximately 30 s. No additional rest or recovery interval was scheduled beyond this brief questionnaire-administration interval, and the same procedure was applied across all experimental conditions. The dimensional weights were calculated separately for each flight phase; that is, the 15 pairwise comparisons completed after a given phase were used to derive the six weights for that phase and to compute the corresponding weighted NASA-TLX score. Let *r_i_* denote the raw rating of the *i*-th dimension and *w_i_* the number of times that dimension was selected in the pairwise comparisons, where *i* = 1, 2, …, 6 and Σ*_i_w_i_* = 15. The weighted overall workload index was used as the primary metric and was computed as:

(8) $${NASA-TLX}_{weighted}=\frac{\sum_{i=1}^{6}r_{i}w_{i}}{15}.$$

The weighted total score ranges from 0 to 100, with higher scores indicating greater overall workload perceived by the participant.

### 2.6. EEG Recording and Analysis

#### 2.6.1. EEG Recording and Preprocessing

Scalp EEG signals were recorded using a SynAmps 2 amplifier (Compumedics NeuroScan, Charlotte, NC, USA) and a 32-channel electrode cap arranged according to the international 10–20 system. GND served as the ground electrode, and M1 and M2 served as the linked-mastoid reference electrodes. The sampling rate was 512 Hz, the online band-pass filter was 0.1–50 Hz, and all electrode impedances were kept below 5 kΩ. EEG preprocessing was performed in MATLAB 2023b using EEGLAB 14.1.1b. Channels not used for EEG analysis, including electrooculographic and electromyographic channels, were removed, leaving 26 scalp electrodes. Continuous EEG data were filtered using a 0.1–50 Hz finite impulse response band-pass filter and a 48–52 Hz notch filter, and were segmented into 2-s epochs.

Epochs containing obvious nonphysiological signal jumps, large movement artifacts, or prominent electromyographic contamination were rejected. Bad channels were identified based on abnormal amplitude or abnormal spectral characteristics and were interpolated when necessary. After bad-channel interpolation and artifact correction, the EEG data remained referenced to the linked mastoids M1 and M2; no additional average re-referencing was applied. ICA was performed using the extended Infomax algorithm implemented in EEGLAB, and independent components were classified using ICLabel. Components with an ICLabel probability greater than 80% for eye, muscle, heart, line-noise, or channel-noise categories were rejected after confirmation based on scalp topography, time course, and spectral characteristics. Epochs with absolute amplitudes exceeding 100 μV were then rejected [22]. EEG quality-control indices, including interpolated channels, rejected ICA components, rejected epochs, and retained valid epochs, are reported by condition and phase in Supplementary Table S3.

#### 2.6.2. Computation of EEG Power Spectra

Regional power spectral density (PSD) analysis of the preprocessed EEG data was performed in MATLAB 2023b. Scalp electrodes were divided into frontal, central, parietal, and occipital regions according to their positions; the electrode assignments of each region are shown in Table 1. For each simulated flight task condition, the continuous EEG signal of each channel was divided into analysis windows of 2 s in length, with 50% overlap between adjacent windows. The PSD was estimated using Welch’s method: a Hamming window was applied to each analysis window to reduce spectral leakage; the windowed signal was then subjected to a fast Fourier transform (FFT) to obtain the modified periodogram of each window, and the periodograms of all analysis windows within the same task condition were averaged to obtain the PSD estimate of each channel under that condition. The frequency resolution was determined by the sampling rate and the FFT length, which was set to the smallest power of two greater than or equal to the number of samples per window. After the PSD was obtained, the absolute band power was computed by numerically integrating the PSD within each predefined frequency band: delta (1–4 Hz), theta (4–8 Hz), alpha (8–13 Hz), beta (13–30 Hz), and gamma (30–45 Hz). To reduce the skewness of the power distribution and facilitate subsequent statistical comparisons, the absolute band power was converted to decibel (dB) values according to 10log10(P), where P is the linear absolute power of the corresponding band. Analysis windows containing invalid data or residual artifacts were not included in the PSD averaging [22,23].

#### 2.6.3. Resting-State EEG Functional Connectivity Analysis

Resting-state EEG functional connectivity was quantified using the debiased weighted phase lag index (dWPLI). The dWPLI estimates the level of phase synchronization from the consistency of nonzero phase differences between different EEG signals and can, to a certain extent, attenuate the influence of spurious zero-phase connectivity caused by volume conduction and the common reference. A larger dWPLI value indicates a higher degree of phase synchronization between the signals of two electrodes. Based on the preprocessed resting-state EEG data, dWPLI values between all electrode pairs were computed separately within the delta (1–4 Hz), theta (4–8 Hz), alpha (8–13 Hz), beta (13–30 Hz), and gamma (30–45 Hz) bands, constructing a functional connectivity matrix for each participant under each intervention condition and at each time point [24]. Each connectivity matrix is symmetric, and its elements represent the phase synchronization strength of the corresponding electrode pair [25]. The analysis included the sham iTBS and active iTBS conditions, and changes in resting-state functional connectivity from before to after the iTBS intervention were compared between the two conditions.

### 2.7. Exploratory Cross-Modal Analysis: Within-Subject Associations Between Flight Performance Changes and Candidate Task-State EEG Feature Changes

To explore the within-subject correspondence between task-state EEG changes and composite flight performance changes during prolonged simulated flight, an exploratory cross-modal association analysis was conducted. Taking each participant-by-stimulation-condition observation as the analytic unit while accounting for the repeated-measures structure, the endpoint change from the early to the late phase of the task was computed for each participant under each stimulation condition. For any EEG metric or the composite flight performance metric X, the change was defined as ΔXᵢⱼ = Xᵢⱼ, Phase3 − Xᵢⱼ, Phase1, where i denotes the participant and j denotes the stimulation condition. A positive value indicates an increase in the metric from Phase 1 to Phase 3, and a negative value indicates a decrease. The endpoint change (Phase 3 minus Phase 1) was used to summarize the state evolution from the early to the late stage of the sustained flight task; Phase 2 was not included in this exploratory association analysis.

Because task-state EEG can yield numerous combinations of frequency bands, brain regions, and time phases, unconstrained association screening across all metrics would increase the risk of multiple comparisons and spurious findings. This study therefore adopted a theory-driven, limited candidate-feature strategy, selecting frontal theta, frontal alpha, and parietal alpha power changes as candidate EEG features. Frontal theta was regarded as a candidate index related to sustained cognitive control and executive regulation [26]; frontal alpha was regarded as a candidate index related to prefrontal attentional-state regulation [27]; and parietal alpha was regarded as a candidate index related to visuospatial attention, sensory information gating, and visual monitoring [28]. The above processes are closely related to instrument monitoring, spatial information integration, goal maintenance, and continuous operational correction during prolonged simulated flight.

For each candidate EEG feature, linear models containing participant fixed effects and stimulation-condition fixed effects were fitted with the EEG endpoint change and the composite flight performance endpoint change as dependent variables, respectively, and the residuals were extracted. Pearson correlation coefficients were then computed between the residualized EEG endpoint changes and the residualized composite flight performance endpoint changes. This analysis estimates the within-subject residual association between EEG changes and performance changes after controlling for stable participant differences and the average effects of stimulation condition.

The EEG spectral analyses were exploratory in nature. Statistical significance for the EEG–behavior associations was assessed using within-subject permutation tests. In each permutation, the labels of the EEG endpoint changes across stimulation conditions were randomly reassigned within each participant, and the residualization and correlation computations were then repeated to obtain a null distribution. Permutation *p*-values were two-tailed. Because these analyses focused on a predefined set of candidate EEG spectral features, multiple-comparison correction was applied locally within this candidate-feature set rather than across all possible electrodes, frequency bands, and endpoints. The permutation *p*-values of the three candidate features were corrected for the false discovery rate (FDR) using the Benjamini–Hochberg method, and associations with FDR-corrected q < 0.05 were considered to survive this local correction. These exploratory analyses were not used to infer causal relationships, mediating mechanisms of the stimulation effects, or predictive validity of EEG in flight performance.

### 2.8. Statistical Analysis

Data analysis was performed under a blinded protocol: all behavioral and EEG datasets were recoded upon export to ensure that analysts remained unaware of the intervention conditions until the final statistical results were produced.

#### 2.8.1. Flight Performance Analysis

Flight performance was analyzed with the composite flight performance standardized score of each flight phase as the dependent variable, using a 3 × 3 within-subject repeated-measures ANOVA in which intervention condition (active iTBS, sham iTBS, and no-stimulation control) and flight phase (Phase 1, Phase 2, and Phase 3) were within-subject factors, with emphasis on testing the interaction between intervention condition and flight phase. If the interaction was significant, simple effects analyses with Bonferroni correction were further conducted. If Mauchly’s sphericity test indicated that the sphericity assumption was not satisfied, Greenhouse–Geisser correction was applied.

Bootstrap confidence intervals were computed from participant-level paired differences. For each comparison, 10,000 bootstrap resamples were drawn with replacement from the participant-level paired differences, and percentile-based 95% confidence intervals were calculated from the empirical bootstrap distribution. The random seed was set to rng (20260729) to ensure reproducibility.

To assess the robustness of the CPS findings under the crossover design, three linear mixed-effects sensitivity models were additionally fitted for CPS. Model 1 included Condition, Phase, their interaction, Period, and Sequence as categorical fixed effects, with Subject modeled as a random intercept: CPS ~ Condition × Phase + Period + Sequence + (1|Subject).

Model 2 examined simple carryover-related effects by adding the immediately preceding stimulation condition as a fixed effect: CPS ~ Condition × Phase + Period + Sequence + PreviousCondition + (1|Subject). Because the immediately preceding condition was undefined in Period 1, Model 2 was fitted using data from Periods 2 and 3 only. PreviousCondition included three categorical levels: active iTBS, sham stimulation, and no-stimulation.

Model 3 added the Condition × Period interaction to examine whether the condition effect varied across experimental periods: CPS ~ Condition × Phase + Period + Sequence + Condition × Period + (1|Subject). These models were conducted as sensitivity analyses and were not intended to replace the prespecified repeated-measures ANOVA or to definitively exclude all possible carryover-related effects. Full model specifications and results are provided in Supplementary Table S2.

#### 2.8.2. NASA-TLX Scale Analysis

Subjective workload was analyzed with the NASA-TLX score of each flight phase as the dependent variable, using a 3 × 3 within-subject repeated-measures ANOVA in which intervention condition (active iTBS, sham iTBS, and no-stimulation control) and flight phase (Phase 1, Phase 2, and Phase 3) were within-subject factors, with emphasis on examining the interaction between intervention condition and flight phase. If the interaction was significant, simple effects analyses with Bonferroni correction were further conducted. If Mauchly’s sphericity test indicated that the sphericity assumption was not satisfied, Greenhouse–Geisser correction was applied.

#### 2.8.3. EEG Analysis

##### Resting-State Power Spectrum Analysis

With band power as the dependent variable, a 2 × 2 within-subject repeated-measures ANOVA was conducted in which intervention condition (active iTBS, sham iTBS) and time point (pre-iTBS intervention, post-iTBS intervention) were within-subject factors. If the interaction between intervention condition and time point was significant, simple effects analyses with Bonferroni correction were further conducted.

##### Resting-State Functional Connectivity Analysis

With the dWPLI value of each individual electrode-pair connection as the dependent variable, a 2 × 2 within-subject repeated-measures ANOVA was conducted separately within each frequency band. Intervention condition (active iTBS, sham iTBS) and time point (pre-iTBS intervention, post-iTBS intervention) were both set as within-subject factors, with emphasis on testing the interaction between intervention condition and time point. When the interaction between intervention condition and time point reached the significance level, simple effects analyses were performed to compare pre- versus post-intervention dWPLI changes under each intervention condition, as well as differences between the active iTBS and sham iTBS conditions at each time point. Post hoc pairwise comparisons for simple effects were corrected using the Bonferroni method. Given that edge-level functional connectivity analysis involves repeated testing of a large number of electrode pairs, the Benjamini–Hochberg false discovery rate correction was applied to the interaction test results of all individual connections within each frequency band to control the false-positive risk caused by multiple comparisons. After FDR correction, q < 0.05 was adopted as the criterion for statistical significance. For repeated-measures ANOVAs in which the sphericity assumption was not satisfied, Greenhouse–Geisser-corrected results are reported.

##### Task-State Power Spectrum Time-Course Analysis

With the alpha-, beta-, and theta-band power of each brain region as dependent variables, a 3 × 3 within-subject repeated-measures ANOVA was conducted in which intervention condition and flight phase were within-subject factors, with emphasis on examining the interaction between intervention condition and flight phase. If the interaction was significant, simple effects analyses with Bonferroni correction were further conducted.

## 3. Results

### 3.1. Flight Performance

**Figure 3:**
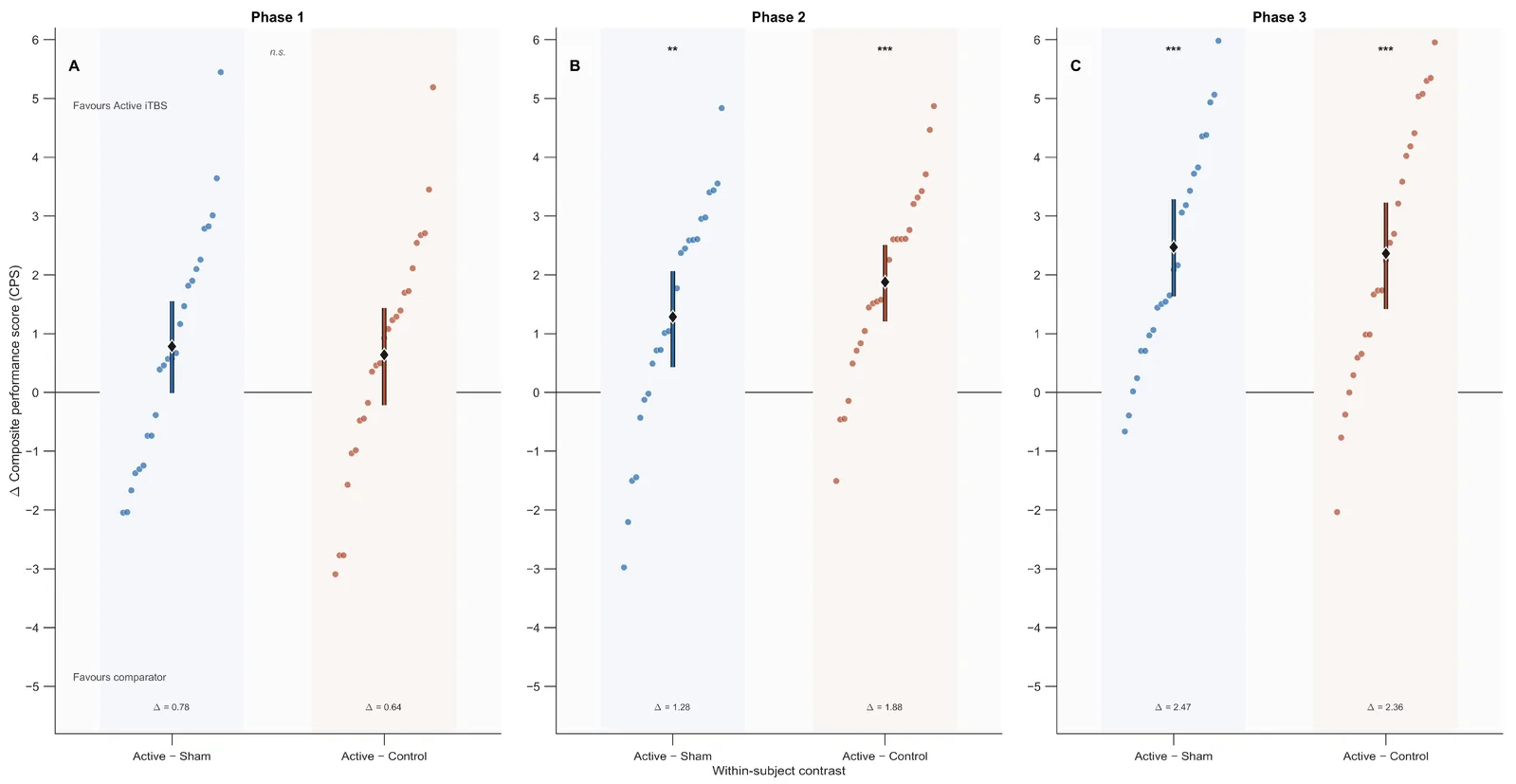
Within-subject effects of active iTBS on composite performance score (CPS) during the simulated flight task. (**A**) Phase 1: paired differences in CPS between active iTBS and the two comparator conditions. (**B**) Phase 2: paired differences in CPS between active iTBS and the two comparator conditions. (**C**) Phase 3: paired differences in CPS between active iTBS and the two comparator conditions. Each point represents the paired difference in CPS for one participant, with positive values favoring active iTBS. Black diamonds indicate mean paired differences, and vertical intervals represent percentile-based 95% confidence intervals derived from 10,000 bootstrap resamples of within-subject paired differences. Numerical labels below each comparison denote the exact mean differences. Active iTBS was associated with significantly higher CPS than both comparator conditions during Phases 2 and 3. Exact confidence interval bounds are provided in Supplementary Table S4. ** *p* < 0.01, *** *p* < 0.001, n.s., not significant. Corresponding Cohen’s d_z_ values are reported in Supplementary Table S4.

**Figure 4:**
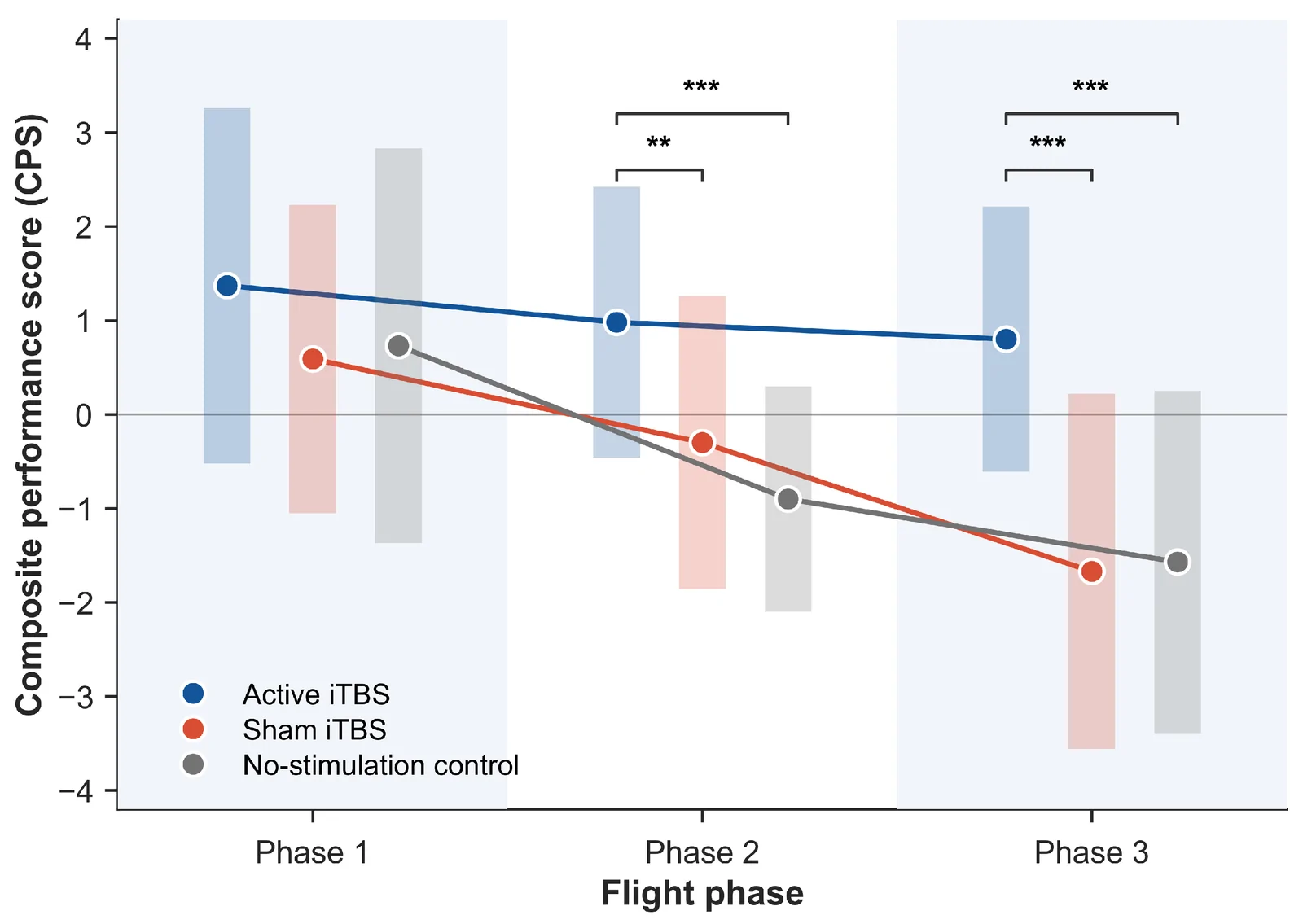
Changes in composite performance score across the 60-min simulated flight task. Points represent group means, and thick vertical intervals represent standard deviations. Lines connect phase-specific group means. Active iTBS was associated with higher CPS than sham iTBS and the no-stimulation control condition in Phases 2 and 3; ** *p* < 0.01, *** *p* < 0.001; CPS, composite performance score; iTBS, intermittent theta burst stimulation.

Performance time-course analysis: the 3 × 3 repeated-measures ANOVA revealed a significant intervention condition × phase interaction (F(4, 96) = 3.656, *p* = 0.007, ηp^2^ = 0.132). Post hoc pairwise comparisons showed that CPS under active iTBS was higher than that under sham iTBS (*p* = 0.006) and the no-stimulation control (*p* < 0.001) in Phase 2, and higher than both comparators in Phase 3 (both *p* < 0.001) (Table 2, Figure 3 and Figure 4).

To assess the robustness of the primary CPS findings to crossover-design factors, we conducted the prespecified sensitivity mixed-effects analyses described above. In Model 1, the Condition × Phase effect remained statistically significant after adjustment for Period and Sequence, F(4, 96) = 3.42, *p* = 0.011. Neither Period, F(2, 48) = 0.84, *p* = 0.438, nor Sequence, F(2, 22) = 0.57, *p* = 0.574, was statistically significant. In Model 2, which was fitted using data from Periods 2 and 3 only, PreviousCondition was not statistically significant, F(2, 136) = 0.69, *p* = 0.503. In Model 3, the Condition × Period interaction was not statistically significant, F(4, 92) = 0.91, *p* = 0.462. These findings support the robustness of the primary CPS result to the assessed period-, sequence-, and simple carryover-related factors; however, they do not definitively exclude all possible crossover-related influences. Full results are reported in Supplementary Table S2.

### 3.2. NASA-TLX Scale Results

**Figure 5:**
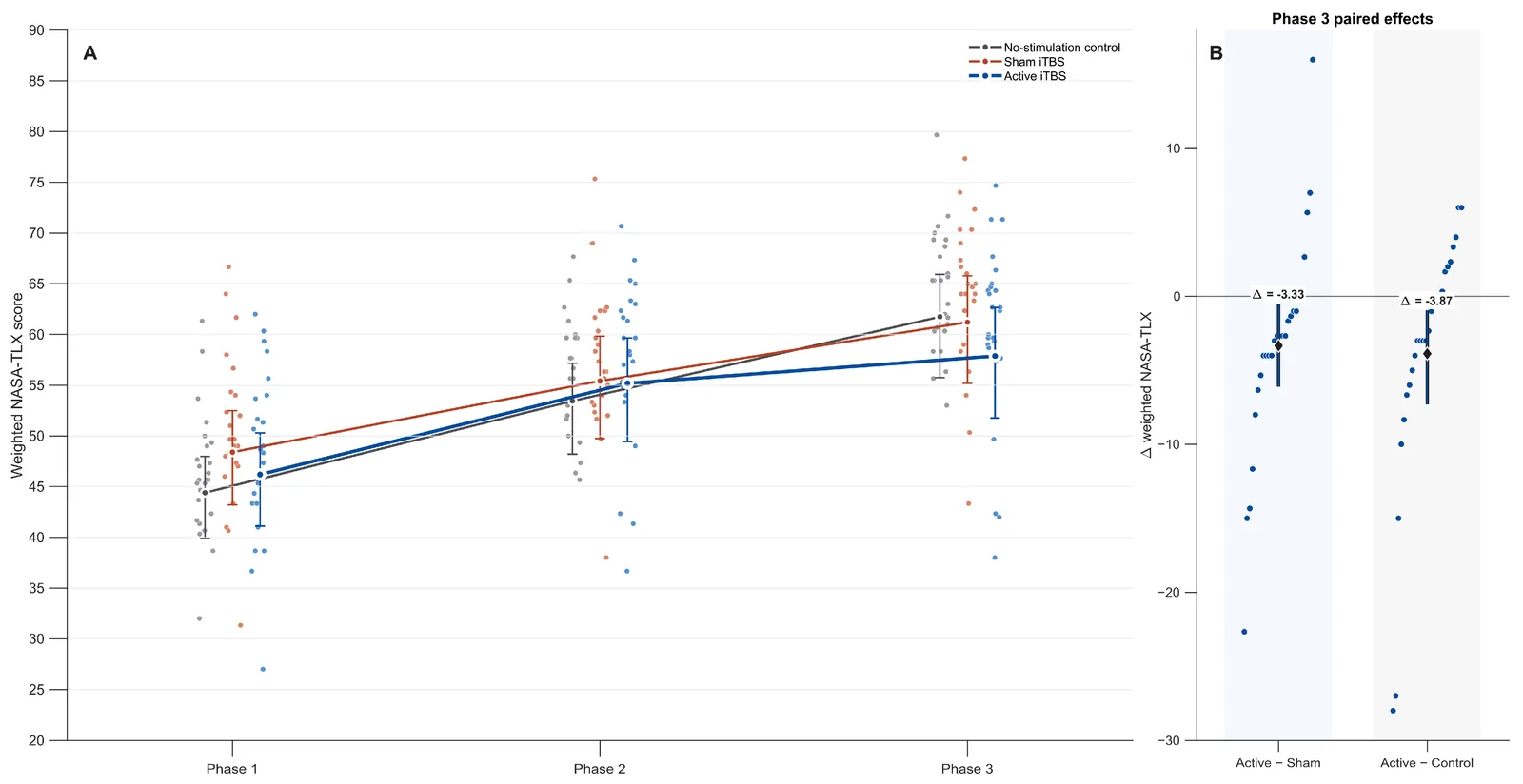
Subjective workload across simulated-flight phases under different stimulation conditions. (**A**) Weighted NASA-TLX scores under no-stimulation control, sham iTBS, and active iTBS. Dots represent individual observations; lines and circles represent means; error bars represent 95% bootstrap confidence intervals of the mean. Exact mean paired differences and confidence intervals for the Phase 3 paired effects are reported in Supplementary Table S5. (**B**) Within-participant Phase 3 differences between active iTBS and each comparator (Active − comparator). Dots represent individual differences, diamonds represent mean differences, and vertical bars indicate bootstrap 95% confidence intervals; negative values indicate lower subjective workload under active iTBS. Corresponding Cohen’s d_z_ values are reported in Supplementary Table S5.

The 3 × 3 repeated-measures ANOVA revealed a significant condition × phase interaction (F(3.693, 88.636) = 3.312, *p* = 0.015, ηp^2^ = 0.121; Greenhouse–Geisser corrected). Post hoc pairwise comparisons showed that NASA-TLX scores in Phase 1 were significantly higher under the sham condition than under the no-stimulation condition (*p* = 0.004); in Phase 3, NASA-TLX scores under the active iTBS condition were significantly lower than those under the sham condition (*p* = 0.038) and the no-stimulation condition (*p* = 0.041) (Table 3, Figure 5). In addition, the main effect of phase was significant: as the simulated flight progressed, NASA-TLX scores in every group were significantly higher in each phase than in the preceding phase (*p* < 0.001). This indicates that subjective workload increased continuously as the flight progressed and was lower under active iTBS in the final phase.

### 3.3. Post-Stimulation, Pre-Task Resting-State EEG

#### 3.3.1. Resting-State EEG Power Spectrum Results

**Figure 6:**
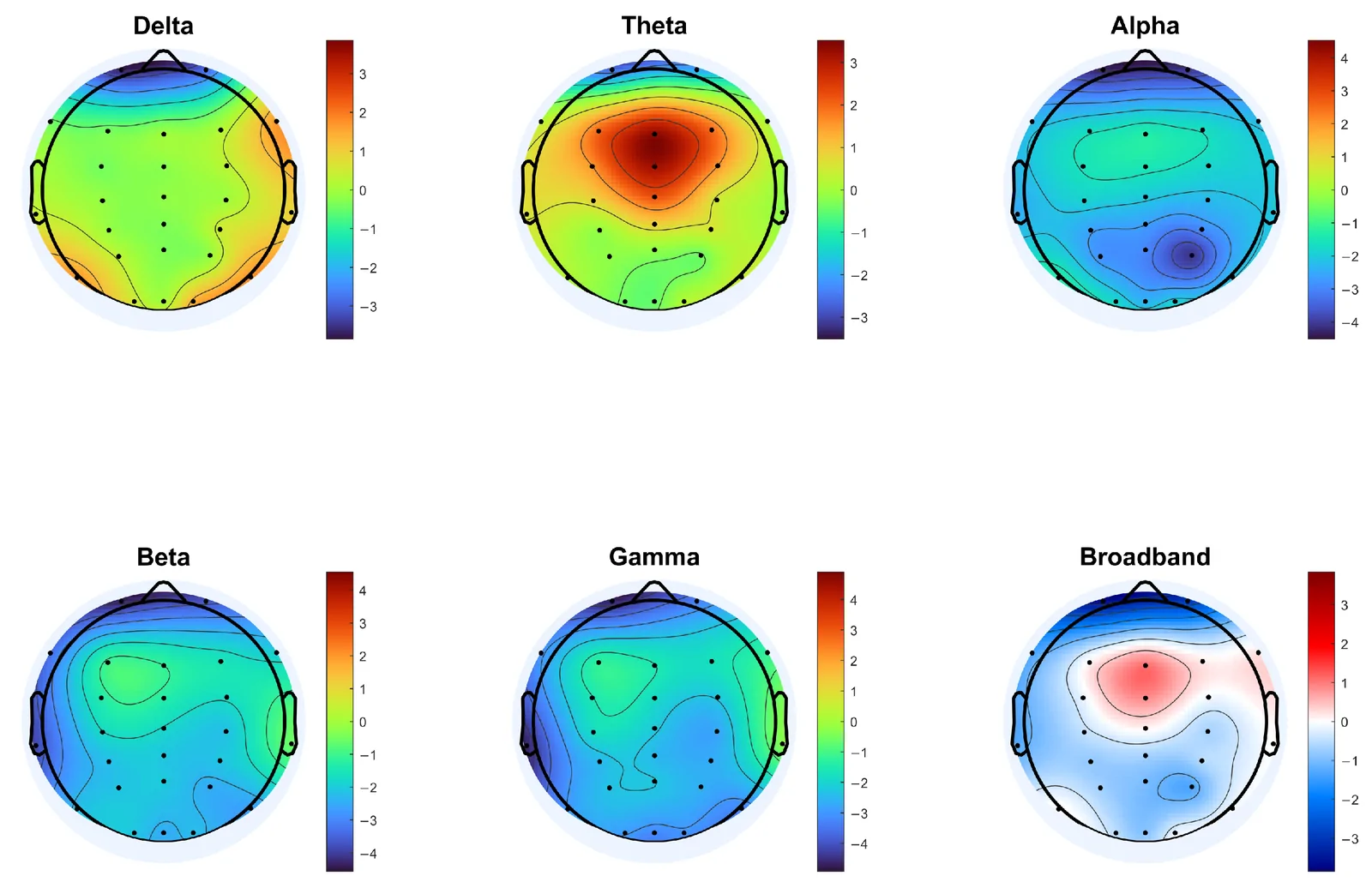
Topographic maps of the 2 × 2 repeated-measures differences before and after intervention under the active iTBS and sham iTBS conditions, computed as (Post–Pre under active iTBS)–(Post–Pre under sham iTBS).

The 2 × 2 repeated-measures ANOVAs on power in each frequency band before and after intervention showed no significant intervention condition × time interaction in the delta band (*p* = 0.410), and the topographic maps revealed no obvious changes in whole-brain power distribution in either group, suggesting that iTBS did not modulate low-frequency slow-wave activity. In the theta band, power showed a slight, non-significant increase after active iTBS (interaction *p* = 0.166); the topographic maps showed a slight expansion of the warm-colored frontal theta region after active iTBS, but this trend did not reach statistical significance and should be interpreted with caution. Significant intervention condition × time interaction effects were detected in the alpha, beta, and gamma bands (all *p* < 0.05); alpha-, beta-, and gamma-band power decreased significantly after intervention only under the active iTBS condition, whereas power under the sham condition changed little (Table 4, Figure 6). These post-stimulation changes in alpha, beta, and gamma power may reflect condition-related alterations in resting-state cortical state or network dynamics. Reduced resting-state alpha power has been associated in some contexts with lower cortical inhibition or higher alertness; however, this association is indirect and context dependent. Likewise, the observed beta and gamma power changes cannot be interpreted here as direct evidence of improved cognitive control or a specific neural mechanism. Because cortical arousal, inhibitory function, task-load processing capacity, and related neurochemical processes were not directly measured, these resting-state EEG differences should be regarded as exploratory neurophysiological observations rather than evidence that iTBS improved baseline cognitive state or established a specific neural mechanism.

#### 3.3.2. Resting-State Functional Connectivity Results Before and After iTBS Intervention Under the Active iTBS and Sham iTBS Conditions

**Figure 7:**
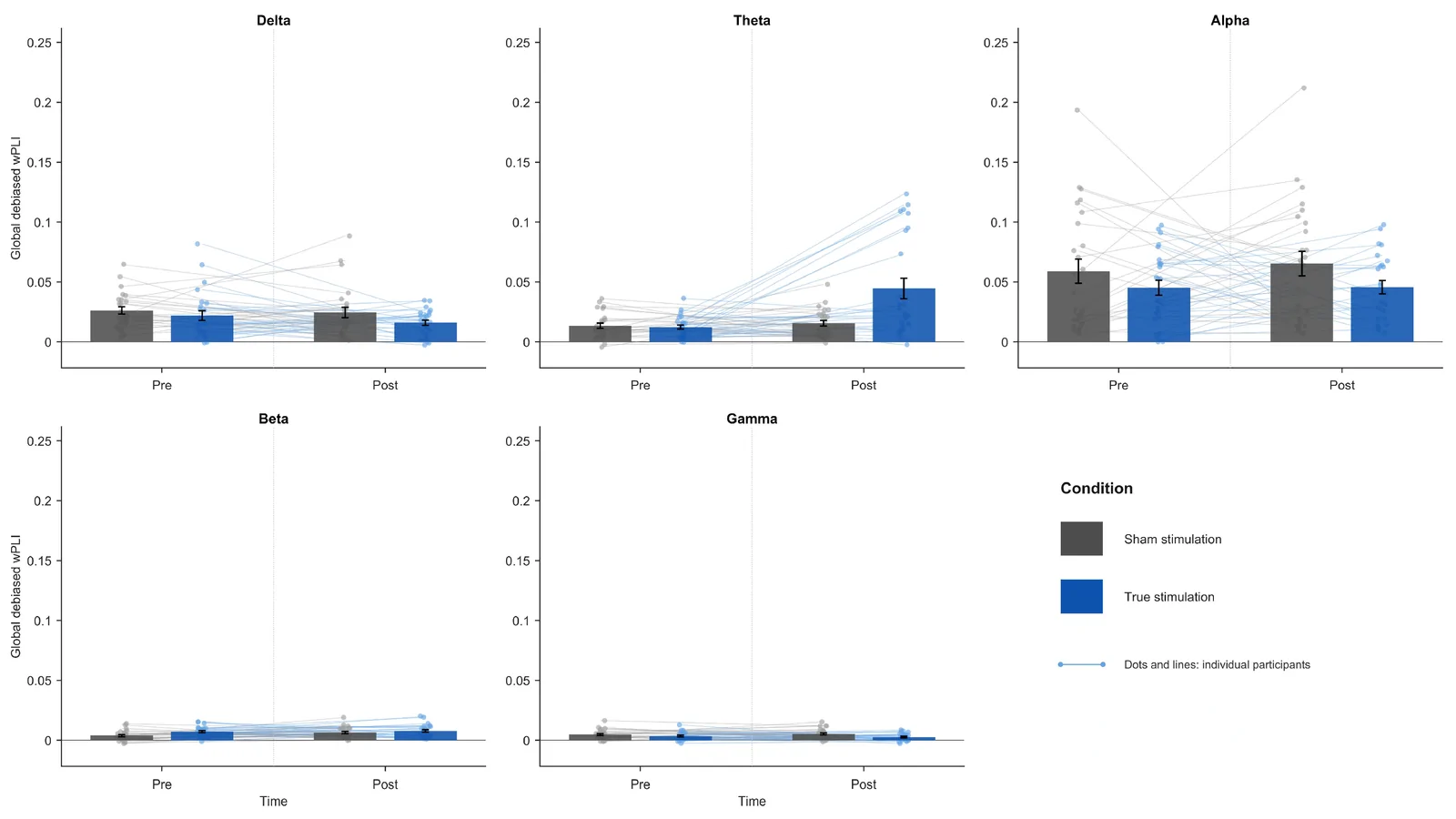
Functional connectivity changes in each frequency band before and after intervention under the active iTBS and sham iTBS conditions.

**Figure 8:**
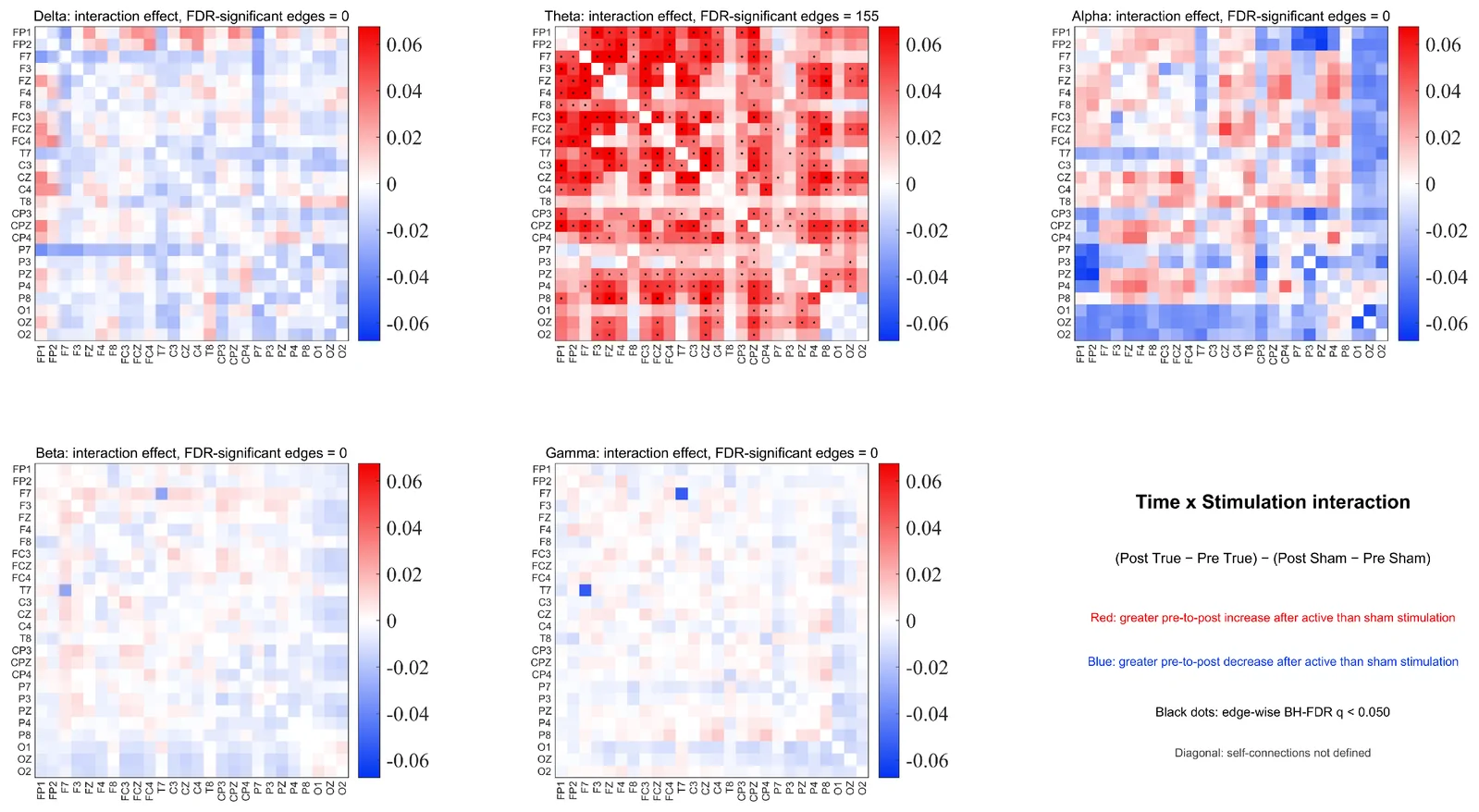
Results of the 2 × 2 repeated-measures ANOVA on functional connectivity in each frequency band before and after intervention under the active iTBS and sham iTBS conditions.

The 2 × 2 repeated-measures ANOVA showed that the modulation of resting-state EEG phase synchronization by iTBS intervention exhibited marked frequency-band specificity. In the theta band (4–8 Hz), a significant intervention condition × time interaction was observed (*p* = 0.003), manifested as a significant enhancement of the dWPLI in 155 individual connections after active iTBS (all FDR q < 0.05), spanning frontal, central, parietal, and temporal regions, whereas no comparable changes were observed under the sham condition (Figure 7 and Figure 8). In contrast, no significant interaction effects were observed in the delta, alpha, beta, or gamma bands (all *p* > 0.05), suggesting that the iTBS intervention did not modulate cortical phase synchronization in these bands. This finding complements the resting-state power results: theta-band phase synchronization was enhanced in the absence of a significant increase in theta power, suggesting that iTBS may modulate low-frequency temporal coordination across brain regions rather than local oscillatory amplitude. Because this study relied on connectivity analysis at the scalp EEG sensor level, the cortical sources of this change, the direction of information flow, and its direct causal relationship with the stimulation rhythm cannot yet be determined.

### 3.4. Task-State Regional EEG Power Spectrum Results

**Figure 9:**
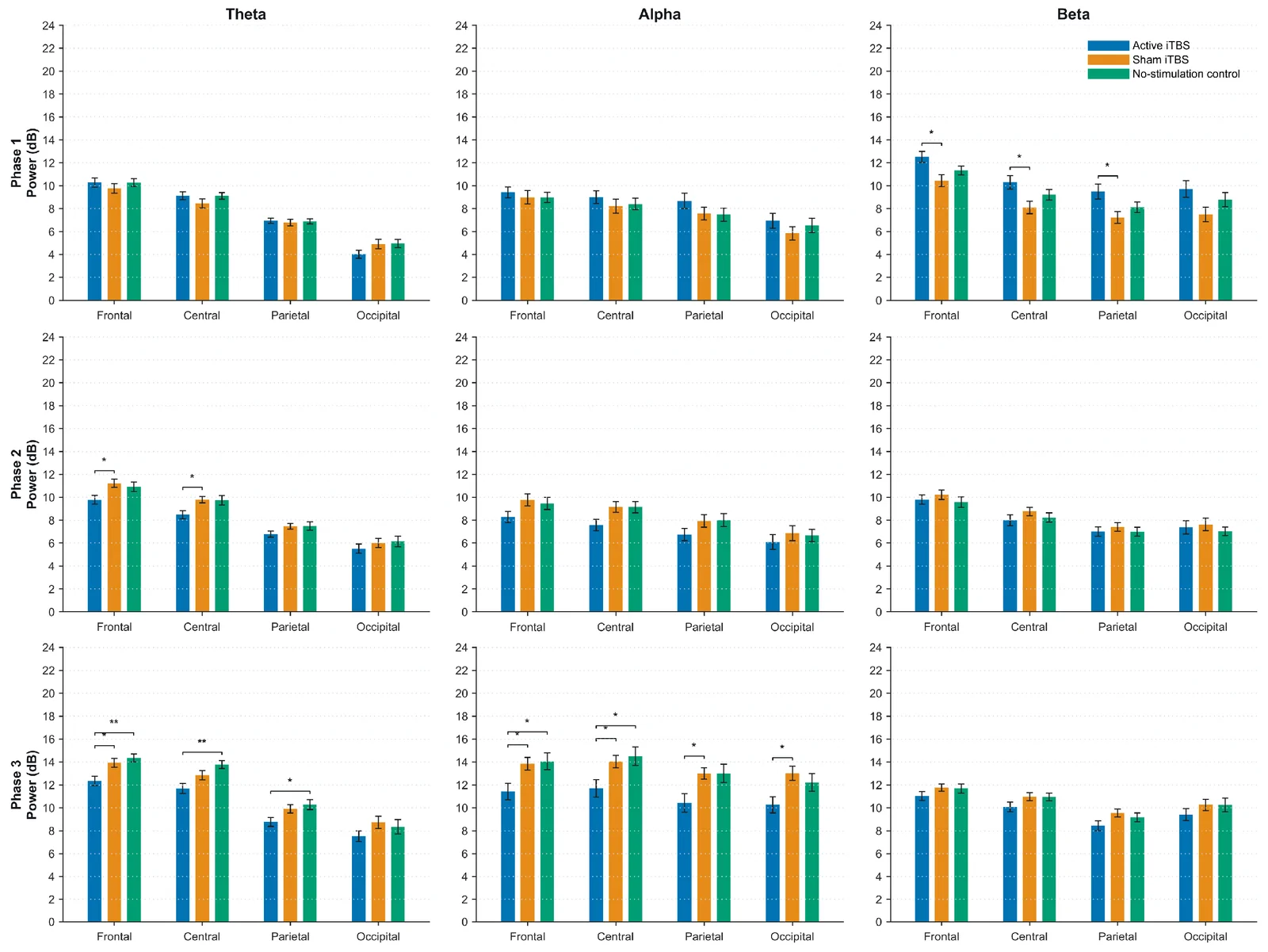
Theta-, alpha-, and beta-band power in the frontal, central, parietal, and occipital regions in Phase 1, Phase 2, and Phase 3, respectively. Blue bars represent the active iTBS condition, orange bars represent the sham iTBS condition, and green bars represent the no-stimulation control condition. Bars represent means, and error bars represent standard errors of the mean; the vertical axis shows EEG power (dB). Within each phase, frequency band, and brain region, Bonferroni correction was applied to the three pairwise comparisons. Brackets indicate between-group differences that were statistically significant after Bonferroni correction; * *p* < 0.05, ** *p* < 0.01. (Details of the significant pairwise differences in EEG spectral power are provided in Table A1 in Appendix A; frequency-specific topographic maps are provided in Figure A1, Figure A2 and Figure A3 in Appendix B).

A 3 × 3 (Condition [active iTBS, sham iTBS, no-stimulation control] × Phase [1, 2, 3]) repeated-measures ANOVA was performed on the power values (dB) of each frequency band in each brain region (Figure 9). To examine how task-state EEG activity changed over the course of the prolonged simulated flight under different stimulation conditions, theta-, alpha-, and beta-band power in the frontal, central, parietal, and occipital regions was compared. The results showed that the EEG effects of active iTBS were clearly phase-dependent: only localized high-frequency activity differences were observed in the early phase of the task, whereas the middle and late phases were dominated by differences in low-frequency power—specifically, relative reductions in frontocentral theta and fronto-parieto-occipital alpha power.

#### 3.4.1. Core Low-Frequency Results in the Middle and Late Phases of the Task

In Phase 2, theta power in the frontal and central regions under the active iTBS condition was lower than that under the sham iTBS condition (both *p* < 0.05). By Phase 3, these low-frequency differences were further expressed as follows: frontal theta power under the active iTBS condition was lower than that under the sham iTBS and no-stimulation conditions, and central theta power was lower than that under the no-stimulation condition; meanwhile, alpha power in the frontal, parietal, and occipital regions under the active iTBS condition was lower than that under the sham iTBS condition (all *p* < 0.05).

From the perspective of the task process, theta and alpha power in multiple brain regions showed an increasing trend in the late phase of the task under the sham iTBS and no-stimulation conditions, suggesting that low-frequency oscillatory activity intensified as the task continued and load accumulated. In contrast, no comparable late-phase buildup of low-frequency power was observed under the active iTBS condition. These results indicate that MRI-navigated left-DLPFC active iTBS may modulate time-on-task-related changes in low-frequency neural oscillations during prolonged simulated flight, with its effects emerging mainly in the middle and late phases of the task.

#### 3.4.2. Localized High-Frequency Changes in the Early Phase of the Task

In Phase 1, beta power in parts of the frontal and parietal regions under the active iTBS condition was higher than that under the sham iTBS condition. Because these high-frequency differences were largely confined to the early phase of the task and did not show the same cross-phase pattern as the later low-frequency power changes, they are regarded as localized, exploratory stimulation-related EEG changes. 

#### 3.4.3. Summary of Task-State EEG Results

In summary, the task-state EEG effects of active iTBS did not manifest as generalized changes across frequency bands and brain regions but mainly as differences in low-frequency activity patterns in the middle and late phases of the prolonged task: frontocentral theta and fronto-parieto-occipital alpha power were relatively lower under the active iTBS condition. This temporal pattern is consistent with the higher composite flight performance exhibited under the active iTBS condition in the middle and late phases of the task and the lower subjective workload reported at the end of the task. The above findings support the notion that active iTBS may influence the evolution of neural states during sustained high-load tasks; accordingly, we further investigated the within-subject associations between flight performance changes and candidate task-state EEG feature changes.

### 3.5. Within-Subject Associations Between Flight Performance Changes and Candidate Task-State EEG Feature Changes

**Figure 10:**
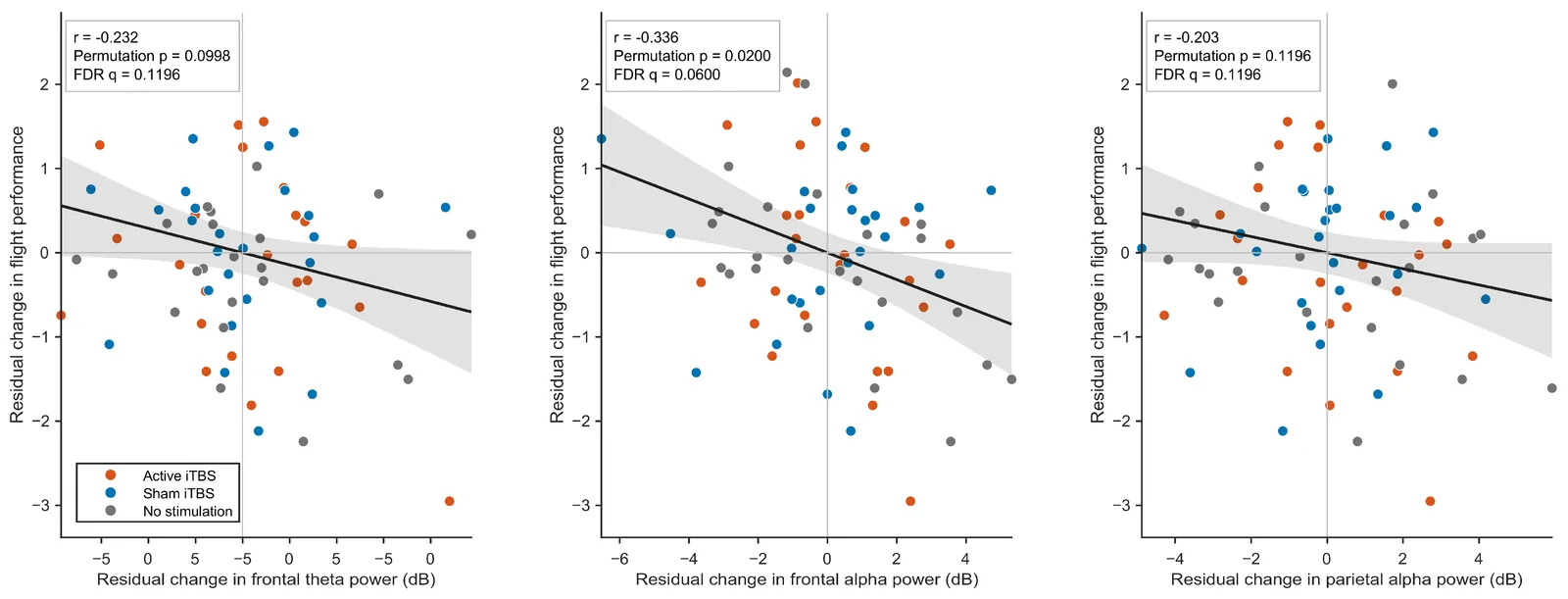
Residual associations between changes in flight performance and EEG power for the three pre-specified candidate predictors. Changes in flight performance and EEG were defined as Phase 3 minus Phase 1. Correlation coefficients were calculated from residuals after controlling for participant and stimulation-condition fixed effects. For the permutation test, EEG endpoint-change labels were randomly reshuffled within each participant’s three conditions; fixed effects were refitted and residuals recalculated before the correlation coefficient was estimated for each permutation. FDR correction was restricted to frontal theta power, frontal alpha power, and parietal alpha power. Black solid lines represent fitted linear regression lines, and grey shaded bands represent 95% confidence intervals.

To explore the within-subject correspondence between task-state EEG changes and composite flight performance changes, the endpoint change from Phase 1 to Phase 3 was computed for each participant × stimulation condition observation, and residual correlation analyses were performed after controlling for participant fixed effects and stimulation-condition fixed effects. All three theory-driven candidate EEG features showed negative association trends with composite flight performance changes (Figure 10). Among them, the association was largest for frontal alpha power change (*r* = −0.336, permutation *p* = 0.0200, FDR-corrected *q* = 0.0600). The association between frontal theta power change and performance change was *r* = −0.232 (permutation *p* = 0.0998, FDR-corrected *q* = 0.1196), and the association between parietal alpha power change and performance change was *r* = −0.203 (permutation *p* = 0.1196, FDR-corrected *q* = 0.1196). None of the three associations reached statistical significance after FDR correction. Therefore, although the directions of the associations were consistent, the results only suggest that within-subject covariation may exist between candidate EEG power changes and flight performance changes, and no definitive associative or mechanistic inference can be drawn from them.

## 4. Discussion

This study adopted a randomized, order-balanced, within-subject crossover design to investigate the effects of MRI-navigated intermittent theta burst stimulation over the left dorsolateral prefrontal cortex on task performance, subjective workload, and EEG responses during sustained simulated flight. The results showed that the effects of active iTBS were markedly time-dependent: compared with the sham stimulation and no-stimulation reference conditions, active iTBS was associated with better composite flight performance mainly in the middle and late phases of the task; at the end of the task, NASA-TLX scores under the active iTBS condition were also lower. Concurrently, resting-state EEG power and phase synchronization showed band-specific changes after iTBS, while task-state EEG was characterized by relative reductions in frontocentral theta and fronto-parieto-occipital alpha power in the middle and late phases of the task. Although changes in frontal alpha showed a directionally consistent trend with changes in composite performance, the association did not survive FDR correction.

These findings indicate that active iTBS was associated with a phase-dependent behavioral pattern during the simulated flight task. The delayed emergence of the behavioral difference is compatible with state-dependent accounts of non-invasive brain stimulation, in which the observable behavioral consequences of stimulation may depend on the subsequent task context and accumulated cognitive demands. Nevertheless, compatibility with this theoretical account should not be interpreted as direct evidence for LTP-like plasticity [29], improved brain-network efficiency, or a confirmed causal pathway. For neural engineering and human factors research, the present results are best viewed as preliminary evidence that controlled neuromodulation experiments can be combined with simulated operational tasks and EEG recordings to study task-state changes under prolonged workload.

### 4.1. Phase-Dependent Effects of iTBS on Prolonged Simulated Flight Performance

The behavioral advantage of active iTBS emerged mainly in Phases 2 and 3 rather than in Phase 1. Because the target heading, altitude, and airspeed differed across phases, these later-phase differences should not be interpreted as specific evidence that iTBS prevented fatigue-related performance decline. Instead, the findings indicate that, under the sustained simulated-flight task and its phase-specific control requirements, active iTBS was associated with better composite flight performance than sham iTBS and no-stimulation control. The task required continuous monitoring of heading, altitude, and airspeed and continuous corrections based on real-time feedback; therefore, CPS reflects overall control performance under the combined demands of elapsed task time and phase-specific operational requirements.

The left dorsolateral prefrontal cortex is an important node of the cognitive control network and participates in goal-directed behavior, working memory, and attentional resource allocation [30]. The use of MRI navigation for individualized target localization improved the consistency of stimulation sites. The higher composite flight performance in the middle and late phases of the task under the active stimulation condition is consistent with theoretical expectations regarding the involvement of the left dorsolateral prefrontal cortex in sustained cognitive control. It should be noted that this study supports the existence of differences in phase-wise performance patterns across stimulation conditions but did not characterize the slope of performance decay on a continuous time scale. Future studies could further analyze the effects of iTBS on performance fluctuations, the risk of abnormal operations, and trajectories of performance change based on trial-level or short-time-window behavioral data.

### 4.2. iTBS and Subjective Workload

The NASA-TLX results showed that subjective workload exhibited an upward trend under all conditions as the simulated flight task progressed; however, at the end of the task, total NASA-TLX scores under the active stimulation condition were lower than those under the sham stimulation and no-stimulation conditions. This result is directionally consistent with the late-phase performance advantage: under the active stimulation condition, participants did not maintain better control performance by reporting greater subjective effort; instead, they exhibited better late-phase flight performance while perceiving lower workload. This pattern is consistent with the possibility that active iTBS was associated with better late-phase task performance without a concomitant increase in self-reported workload. However, because subjective workload and task performance were assessed under phase-specific task conditions, this finding does not establish a specific neural mechanism or demonstrate that iTBS reduced fatigue-related cognitive cost [31]. From the perspective of aviation ergonomics, this profile of “lower perceived workload alongside maintained performance” is potentially more informative than a simple performance gain; whether it translates into an extended tolerable working time for operators remains to be tested.

### 4.3. Resting-State EEG Spectral Changes: iTBS May Reconfigure the Post-Stimulation Neural State Rather than Simply Increasing Power in a Single Band

The resting-state EEG results showed intervention condition × time interaction effects in alpha-, beta-, and gamma-band power after iTBS, with the beta-band change being the most prominent; theta-band power itself showed no significant condition-related change. In addition, resting-state functional connectivity analysis revealed a significant condition × time interaction in theta-band phase synchronization, with broadly enhanced dWPLI connections after active iTBS but no similar changes under the sham condition.

First, the enhancement of theta phase synchronization without a change in theta power indicates that the effect of iTBS is not a simple increase in local oscillatory energy. A more plausible explanation is that the stimulation altered the temporal coordination of theta rhythms among different brain regions—that is, the stimulation affected phase coupling at the network level rather than power amplitude at a single electrode or a single region [32]. Second, the condition-related changes in alpha, beta, and gamma power indicate that the neural effects produced by iTBS are band-specific. In particular, the beta band is often associated with sensorimotor states, cognitive maintenance, and network stability, but its functional meaning depends strongly on brain region, task state, and analysis metric. Therefore, the beta changes in this study cannot be directly interpreted as “cognitive enhancement” or “increased neural excitability”. A more plausible interpretation is that MRI-navigated DLPFC iTBS induced measurable post-stimulation resting-state neurodynamic changes encompassing both local band power and cross-regional phase synchronization [33]. Although iTBS has been associated with LTP-like modulation of cortical excitability, the present study did not directly assess post-stimulation cortical excitability or synaptic plasticity. Therefore, the observed behavioral and EEG differences cannot be attributed to an LTP-like or LTD-like mechanism.

### 4.4. Task-State EEG: Low-Frequency Power Reduction in the Middle and Late Phases Is a Candidate State Marker, Not an Established Mechanism

In task-state EEG, the differences associated with active iTBS likewise showed phase dependency. In Phase 2, theta power in the frontal and central regions was lower than that under the sham condition; in Phase 3, frontal theta power was lower than that under the sham and no-stimulation conditions, central theta power was lower than that under the no-stimulation condition, and alpha power in the frontal, parietal, and occipital regions was lower than that under the sham condition. Overall, active iTBS was associated with relative reductions in frontocentral theta and fronto-parieto-occipital alpha power in the middle and late phases of the task.

Both the theta and alpha bands are commonly used neuroergonomic indices in prolonged cognitive tasks: frontal theta may be related to cognitive control, conflict monitoring, and compensatory effort [34], whereas alpha may be related to attentional allocation, task engagement, information suppression, and fatigue states [35]. Therefore, the reduction in low-frequency power in this study cannot be simply interpreted as “reduced cognitive load” or “stronger brain function”. Combined with the better late-phase performance and lower NASA-TLX scores, a more cautious interpretation is that active iTBS was associated with a different mode of neural resource allocation during the prolonged task.

The EEG–behavior association analysis provides limited but directionally consistent clues for this interpretation. The change in frontal alpha power was negatively correlated with the change in composite performance, and the permutation test suggested statistical significance; however, this association did not reach significance after FDR correction. In other words, frontal alpha changes may be related to performance improvement, but the current evidence is insufficient to regard it as a reliable biomarker.

### 4.5. EEG–Behavior Associations: Candidate Evidence Rather than an Established Mechanism

The exploratory analysis showed a negative association trend between frontal alpha power change and CPS change (*r* = −0.336, permutation *p* = 0.020, FDR-corrected *q* = 0.060), consistent in direction with the group-level results. However, this association did not survive multiple-comparison correction, and the associations of frontal theta power change and parietal alpha power change with performance change did not reach nominal significance. Therefore, the current data only suggest that a within-subject covariation trend may exist between candidate EEG features and flight performance and are insufficient to establish a predictive relationship or causal mediation. In future studies with larger samples and preregistered designs, multivariable models could be incorporated to validate this effect size.

### 4.6. Implications for Neural Engineering and Human Factors Engineering

This study integrated individualized MRI-navigated stimulation, sustained simulated flight control, objective behavioral performance, subjective workload assessment, and resting-state and task-state EEG measurements. The contribution of this design lies not in demonstrating an immediately deployable intervention protocol for pilots, but in establishing a controlled multimodal laboratory framework for examining relationships among neuromodulation, brain state, workload, and simulated operational behavior.

The present findings should not be interpreted as evidence that iTBS is ready for operational use in aviation or that it improves flight safety. The study did not include professional pilots, real-flight scenarios, emergency events, crew-resource management, or operational safety endpoints. Therefore, any neuroergonomic implication should be limited to hypothesis generation and experimental-framework development. Before applied conclusions can be drawn, future studies should include larger and more diverse samples, trained operators, more ecologically valid flight tasks, emergency-event scenarios, formal blinding assessment, longer follow-up, and direct operational safety outcomes.

### 4.7. Limitations and Future Directions

This study has several limitations. First, the sample consisted of 25 healthy young men; whether the findings generalize to women, trained pilots, older adults, or other operator populations remains unknown. Second, although the simulated flight task afforded good experimental control, it did not reproduce the multisensory demands, unexpected events, emotional stress, crew coordination, or operational constraints of real flight [36], and no direct operational safety outcomes were measured; the observed CPS and NASA-TLX differences should therefore not be interpreted as evidence of improved flight safety or operational efficacy. Third, because the three phases differed in heading, altitude, and airspeed targets, time on task, potential fatigue, and phase-specific control demands are inherently confounded, and their independent contributions cannot be estimated. Fourth, although the 7-day washout, Latin-square balancing, and the crossover sensitivity analyses reduce design-related bias, residual practice, expectancy, period, sequence, or carryover effects cannot be fully excluded; likewise, the brief NASA-TLX administration between phases may have influenced fatigue, attention, or expectancy. Fifth, the CPS is a within-study composite based on internal standardization and equal weighting of the three error metrics, and its absolute values are sample-dependent. Sixth, participant blinding was only partial: the no-stimulation condition was inherently open-label, the sham coil could not fully reproduce the somatosensory experience of active iTBS, and condition guessing was not formally assessed. Moreover, the actual stimulator output and target-position deviation were not systematically archived in a form suitable for precise retrospective reporting and therefore could not be quantified beyond the stimulation settings described in Section 2. Seventh, scalp EEG has limited spatial resolution and cannot identify cortical sources, inter-regional information transfer, or synaptic plasticity; moreover, the task-state EEG results are based on local multiple-comparison correction only and, together with the EEG–behavior associations that did not survive FDR correction, should be regarded as exploratory. Finally, no neurochemical measures were obtained; the observed differences cannot be attributed to dopamine depletion or any other neurotransmitter-related mechanism, and catecholaminergic accounts of compensatory fatigue remain hypotheses to be tested with pharmacological, PET, or MRS designs. Future studies should include larger and more diverse samples, professional operators, more ecologically valid scenarios with emergency events, difficulty-matched task phases, neurochemical measurements, longer follow-up, formal blinding assessment, and direct operational safety endpoints.

## 5. Conclusions

MRI-navigated left-DLPFC iTBS was associated with better maintenance of composite performance and lower subjective workload in the middle and late phases of a prolonged simulated flight task. This effect did not appear at the initial stage of the task but emerged in the middle and late phases, suggesting that the potential role of iTBS may be closer to the modulation of sustained cognitive control and task-state evolution than to a simple enhancement of immediate operational ability. Resting-state EEG showed band-specific changes in power and theta phase synchronization after active iTBS, while task-state EEG showed relative reductions in frontocentral theta and fronto-parieto-occipital alpha power in the middle and late phases. Although the frontal alpha change showed a consistent directional association with performance improvement, this result did not survive FDR correction and therefore cannot be regarded as an established neural mechanism.

Overall, this study established a multimodal neural engineering framework combining MRI-navigated neuromodulation, a simulated flight task, behavioral performance, subjective workload, and EEG state assessment. Future studies should verify the reliability and predictive validity of the candidate EEG features in larger samples using more ecologically valid experimental setups and explore state-dependent closed-loop neuromodulation strategies.

## Figures and Tables

**Table 1:** Brain regions and their electrode assignments.

Brain Region	Electrodes	Number of Electrodes
Frontal	Fp1, Fp2, F7, F3, Fz, F4, F8	7
Central	FC3, FCz, FC4, C3, Cz, C4	6
Parietal	CP3, CPz, CP4, P7, P3, Pz, P4, P8	8
Occipital	O1, Oz, O2	3

Note: T7 and T8 were included only in the whole-brain PSD calculation, not in the regional analysis.

**Table 2:** Flight performance of participants in each phase under the three intervention conditions (M ± SD).

Phase	Active iTBS	Sham iTBS	No-Stimulation Control
Phase 1	1.37 ± 1.89	0.59 ± 1.64	0.73 ± 2.10
Phase 2	0.98 ± 1.44	−0.30 ± 1.56	−0.90 ± 1.20
Phase 3	0.80 ± 1.41	−1.67 ± 1.89	−1.57 ± 1.82

**Table 3:** NASA-TLX scores of participants in each phase under the three intervention conditions (M ± SD).

Phase	Active iTBS	Sham iTBS	No-Stimulation Control
Phase 1	48.04 ± 8.18	50.33 ± 7.55	46.17 ± 6.08
Phase 2	57.39 ± 8.07	57.63 ± 7.32	55.57 ± 5.68
Phase 3	60.19 ± 8.97	63.52 ± 7.51	64.06 ± 5.98

**Table 4:** Resting-state EEG power spectra before and after intervention under the active iTBS and sham iTBS conditions (M ± SD).

Band	Time Point	Active iTBS	Sham iTBS	F (Interaction)	*p* (Interaction)	ηp^2^
δ	Pre	15.10 ± 1.26	14.92 ± 1.36	0.690	0.410	0.028
	Post	14.67 ± 1.58	14.91 ± 1.03			
θ	Pre	10.44 ± 1.62	10.04 ± 1.48	1.979	0.166	0.076
	Post	11.77 ± 2.00	10.43 ± 1.67			
α	Pre	10.41 ± 2.18	10.22 ± 2.18	4.701	0.040	0.164
	Post	8.35 ± 3.29	10.60 ± 2.31			
β	Pre	10.72 ± 0.93	10.10 ± 0.61	38.533	<0.001	0.616
	Post	8.66 ± 1.54	10.48 ± 0.87			
γ	Pre	7.72 ± 1.53	7.31 ± 1.12	24.487	<0.001	0.505
	Post	4.88 ± 1.86	7.32 ± 0.99			

**Figure A1:**
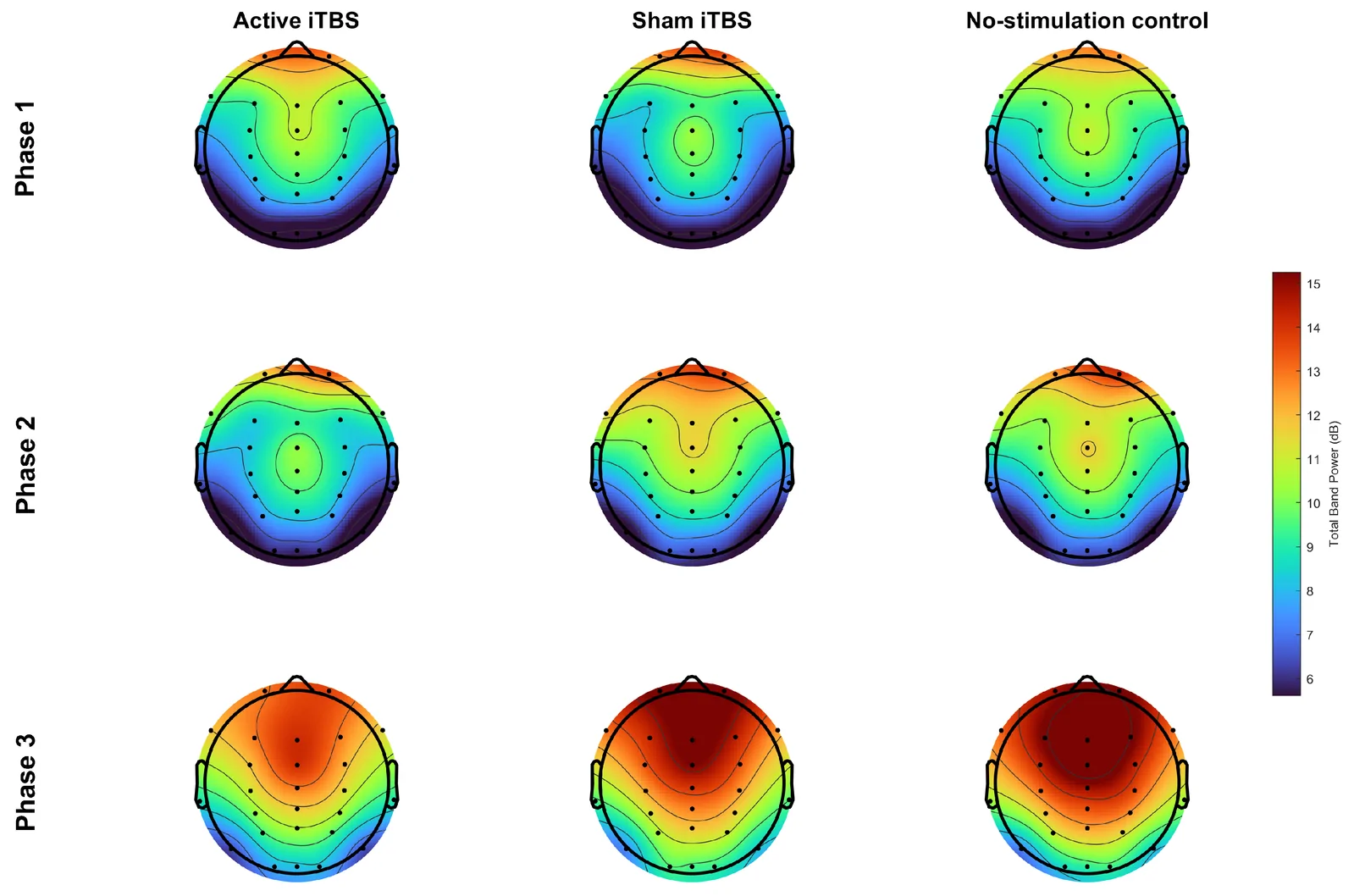
Theta-band EEG topographic maps across intervention conditions.

**Figure A2:**
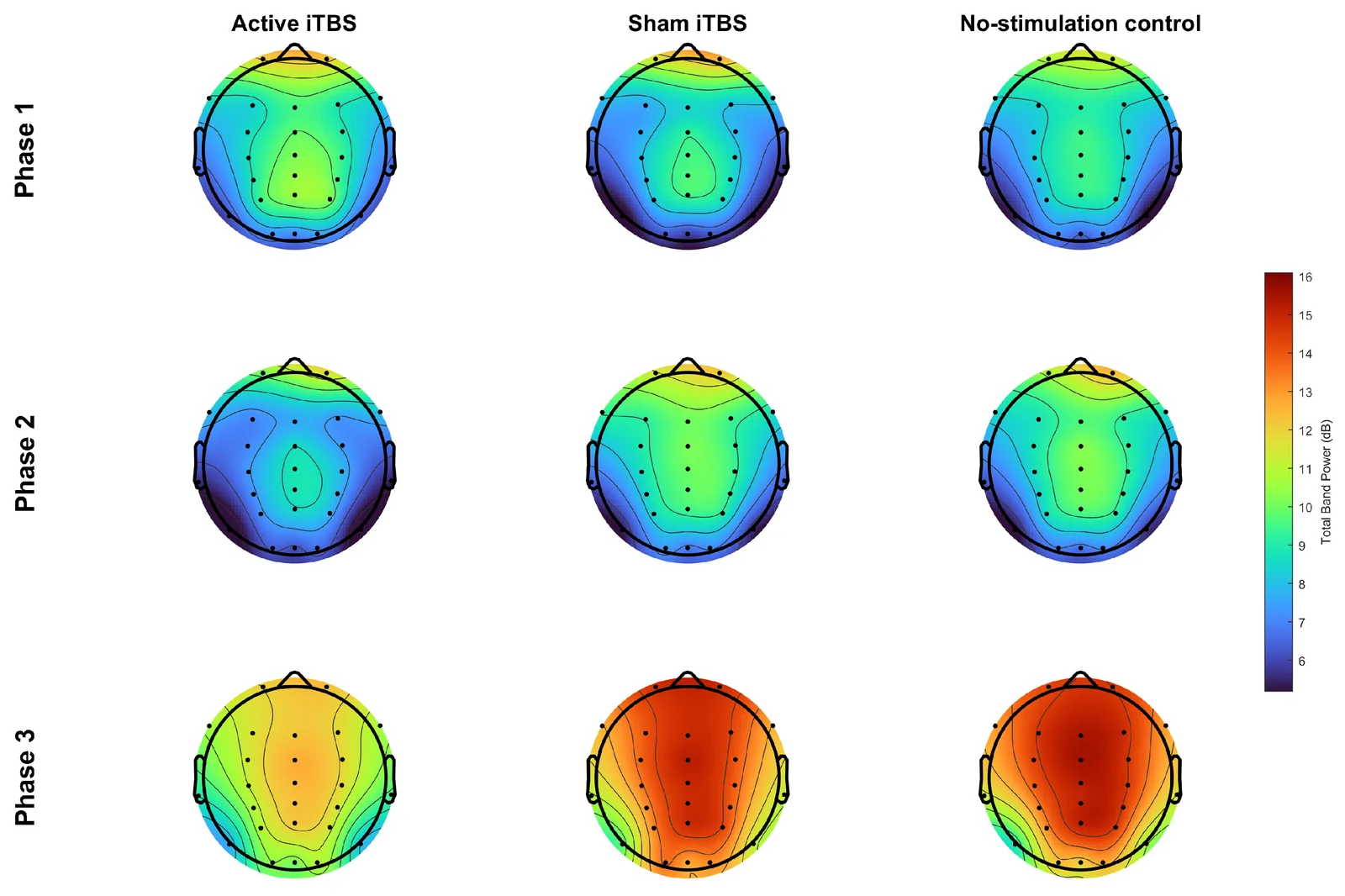
Alpha-band EEG topographic maps across intervention conditions.

**Figure A3:**
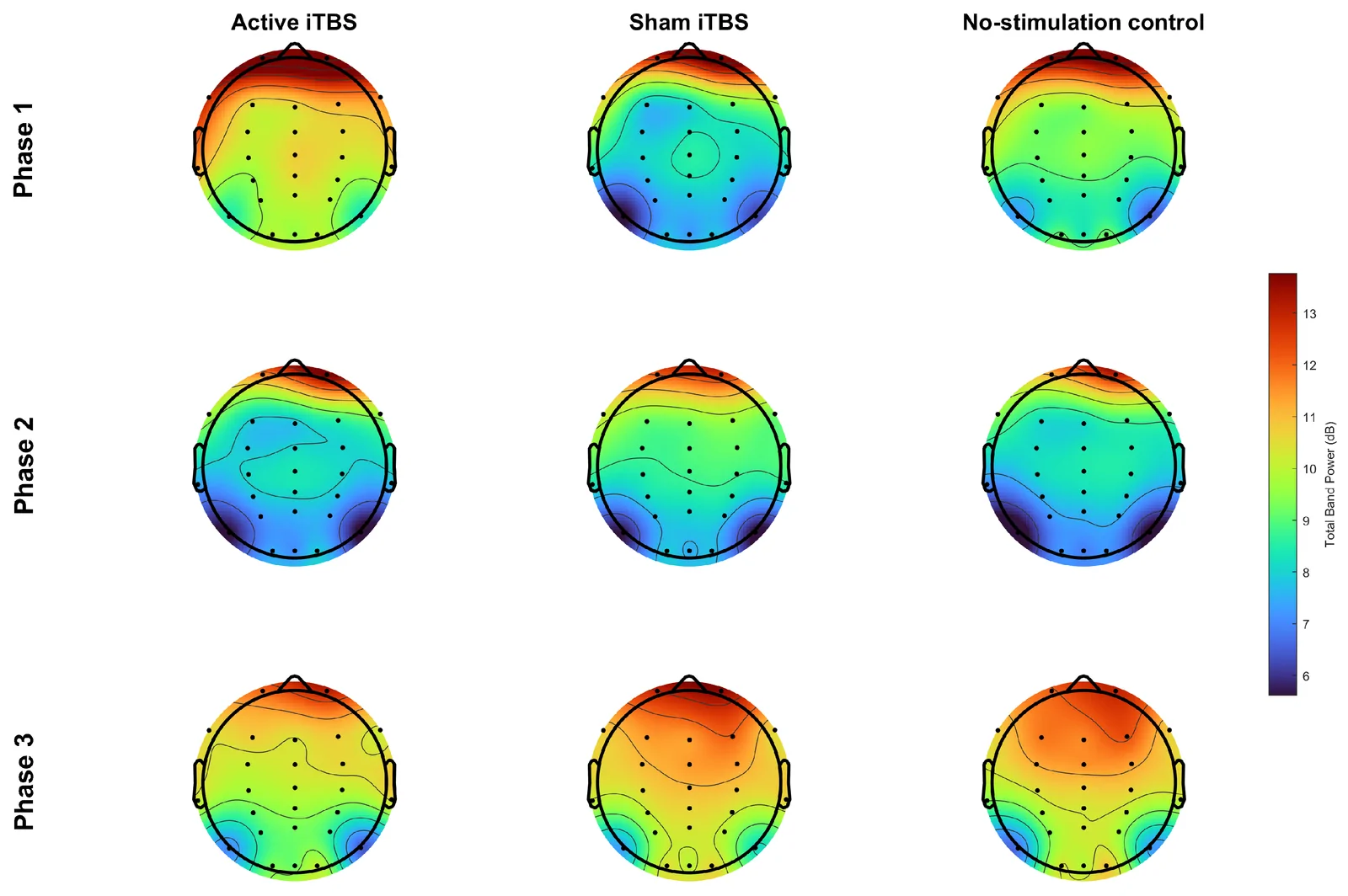
Beta-band EEG topographic maps across intervention conditions.

## Data Availability

Data are available upon reasonable request to the corresponding authors.

## Supplementary Materials

The following supporting information can be downloaded at: https://www.mdpi.com/article/10.3390/bioengineering13090997/s1, Table S1: Assigned treatment sequences and participant allocation in the crossover design; Table S2: Sensitivity mixed-effects analyses for CPS; Table S3: EEG quality-control summary across experimental conditions and flight phases; Table S4: Bootstrapped 95% confidence intervals for mean within-subject paired differences in composite performance score; Table S5: Bootstrapped 95% confidence intervals for the mean paired differences in NASA-TLX.

## Abbreviations

The following abbreviations are used in this manuscript:

CPS	Composite Performance Score
DLPFC	Dorsolateral Prefrontal Cortex
dWPLI	Debiased Weighted Phase Lag Index
EEG	Electroencephalography
iTBS	Intermittent Theta Burst Stimulation
LTD	Long-Term Depression
LTP	Long-Term Potentiation
MEP	Motor-Evoked Potential
MNI	Montreal Neurological Institute
NASA-TLX	NASA Task Load Index
RMSE	Root Mean Square Error
rTMS	Repetitive Transcranial Magnetic Stimulation
TMS	Transcranial Magnetic Stimulation

## Appendix A

**Table A1:** Significant pairwise comparisons of EEG spectral power across brain regions and frequency bands at each task phase.

Phase	Band	Region	Comparison	Mean Difference (dB)	*p* (Bonferroni)	Significance
Phase 1	Beta	Frontal	AC vs. Sham	2.076543279	0.014284387	*
Phase 1	Beta	Central	AC vs. Sham	2.197262016	0.026324816	*
Phase 1	Beta	Parietal	AC vs. Sham	2.265846637	0.024772417	*
Phase 2	Theta	Frontal	AC vs. Sham	−1.445832649	0.02672689	*
Phase 2	Theta	Central	AC vs. Sham	−1.305903192	0.018577238	*
Phase 3	Theta	Frontal	AC vs. Sham	−1.573127913	0.018006086	*
Phase 3	Theta	Frontal	AC vs. NS	−2.010621768	0.001539755	**
Phase 3	Theta	Central	AC vs. NS	−2.089729331	0.001691105	**
Phase 3	Theta	Parietal	AC vs. NS	−1.516249011	0.038640216	*
Phase 3	Alpha	Frontal	AC vs. Sham	−2.437576069	0.031038427	*
Phase 3	Alpha	Frontal	AC vs. NS	−2.640533657	0.030738257	*
Phase 3	Alpha	Central	AC vs. Sham	−2.334028219	0.044204217	*
Phase 3	Alpha	Central	AC vs. NS	−2.809171413	0.040556145	*
Phase 3	Alpha	Parietal	AC vs. Sham	−2.571514508	0.029630325	*
Phase 3	Alpha	Occipital	AC vs. Sham	−2.766682093	0.015016207	*

Note: Only pairwise comparisons with *p* < 0.05 are presented. AC, active iTBS; NS, no stimulation. Mean difference represents the difference in mean EEG spectral power between the two compared groups; a positive value indicates higher spectral power in the first-listed group, whereas a negative value indicates lower spectral power in the first-listed group. * *p* < 0.05; ** *p* <0.01.
