# Supplementary material for: Effects of MRI-Navigated Intermittent Theta Burst Stimulation on Task Performance, Subjective Workload, and EEG Responses During Prolonged Simulated Flight: A Randomized Crossover Study
Source: Bioengineering (Basel). 2026 Aug 27;13(9):997. doi: 10.3390/bioengineering13090997 (PMC13603947; doi:10.3390/bioengineering13090997)
Supplement: Supplementary file 1 [file bioengineering-13-00997-s001.zip › bioengineering-4505900-supplementary.pdf]

Supplementary Table S1. Assigned treatment sequences and participant allocation in the crossover design.

Assigned sequence	Session 1	Session 2	Session 3	Participants
**Sequence 1**	Active iTBS	Sham iTBS	No-stimulation	8
**Sequence 2**	Sham iTBS	No-stimulation	Active iTBS	8
**Sequence 3**	No-stimulation	Active iTBS	Sham iTBS	9

Note. Participants completed all three conditions in a three-sequence Latin-square arrangement. Because the total sample size was 25, allocation across sequences was approximately rather than perfectly balanced

Supplementary Table S2. Sensitivity mixed-effects analyses for CPS

Outcome	Sensitivity model	Term of interest	Test statistic	df	p value	Interpretation
**CPS**	Model 1	Condition × Phase	F = 3.42	(4, 96)	0.011	Condition × Phase effect remained statistically significant
**CPS**	Model 1	Period	F = 0.84	(2, 48)	0.438	No statistically significant period effect
**CPS**	Model 1	Sequence	F = 0.57	(2, 22)	0.574	No statistically significant sequence effect
**CPS**	Model 2	PreviousCondition	F = 0.69	(2, 136)	0.503	No statistically significant PreviousCondition effect
**CPS**	Model 3	Condition × Period	F = 0.91	(4, 92)	0.462	No statistically significant Condition × Period interaction

Note. Values are from sensitivity mixed-effects analyses for the composite flight performance standardized score. Model 1: CPS ~ Condition × Phase + Period + Sequence + (1 ∣ Subject). Model 2: CPS ~ Condition × Phase + Period + Sequence + PreviousCondition + (1 ∣ Subject), fitted using data from Periods 2 and 3 only because the immediately preceding stimulation condition was undefined in Period 1. Model 3: CPS ~ Condition × Phase + Period + Sequence + Condition × Period + (1 ∣ Subject). Condition, Phase, Period, Sequence, and PreviousCondition were treated as categorical fixed effects, and Subject was modeled as a random intercept. These analyses were conducted as sensitivity analyses and were not intended to replace the prespecified repeated-measures ANOVA or to definitively exclude all possible carryover effects.

Supplementary Table S3. EEG quality-control summary across experimental conditions and flight phases.

Condition	Phase	Participants	Interpolated channels	Rejected ICA components	Rejected epochs	Retained epochs	Retention rate
**Active iTBS**	Phase 1	25	2.15±1.12	3.45±1.25	75.32±15.45	524.68±15.45	87.45%±2.58%
**Active iTBS**	Phase 2	25	2.34±1.28	3.82±1.50	82.16±18.30	517.84±18.30	86.31%±3.05%
**Active iTBS**	Phase 3	25	1.98±0.95	3.20±1.10	64.88±12.20	535.12±12.20	89.19%±2.03%
**Sham iTBS**	Phase 1	25	2.45±1.30	4.12±1.45	88.40±20.15	511.60±20.15	85.27%±3.36%
**Sham iTBS**	Phase 2	25	2.52±1.38	4.05±1.32	95.64±22.40	504.36±22.40	84.06%±3.73%
**Sham iTBS**	Phase 3	25	2.28±1.15	3.75±1.28	78.50±16.80	521.50±16.80	86.92%±2.80%
**No-stimulation**	Phase 1	25	1.85±0.88	3.15±1.05	55.20±10.50	544.80±10.50	90.80%±1.75%
**No-stimulation**	Phase 2	25	2.05±1.02	3.30±1.12	62.35±14.15	537.65±14.15	89.61%±2.36%

**No-stimulation**	Phase 3	25	1.76±0.82	2.95±0.90	48.80±9.60	551.20±9.60	91.87%±1.60%

Note： Values are presented as mean ± standard deviation. The table reports the number of interpolated channels, rejected ICA components, rejected EEG epochs, retained valid EEG epochs, and epoch-retention rate for each experimental condition and flight phase.

Supplementary Table S4. Bootstrapped 95% Confidence Intervals for Mean Within-Subject Paired Differences in Composite Performance Score.

Comparison	Task Phase	Mean Difference	95% CI Lower Bound	95% CI Upper Bound	Cohen's d_z
**Active vs. Sham**	Phase 1	0.78	-0.06	1.45	0.41
**Active vs. Sham**	Phase 2	1.28	0.65	1.98	0.75
**Active vs. Sham**	Phase 3	2.47	1.75	3.20	1.34
**Active vs. No-stimulation**	Phase 1	0.64	−0.17	1.35	0.35
**Active vs. No-stimulation**	Phase 2	1.88	1.22	2.65	1.03
**Active vs. No-stimulation**	Phase 3	2.36	1.60	3.15	1.19

Note: Mean differences were calculated as Active iTBS minus the comparison condition. Percentile bootstrap 95% confidence intervals were based on 10,000 resamples of participant-level paired differences.

Supplementary Table S5. Bootstrapped 95% Confidence Intervals for the Mean Paired Differences in NASA-TLX.

Comparison	Task Phase	Mean Difference	95% CI Lower Bound	95% CI Upper Bound	Cohen's d_z
Active − Sham	Phase 3	−3.33	−5.93	−0.81	-0.51
Active − No-stimulation	Phase 3	−3.87	−7.07	−1.02	-0.50

Note: Mean differences were calculated as Active iTBS minus the comparison condition. Percentile bootstrap 95% confidence intervals were based on 10,000 resamples of participant-level paired differences. Only Phase 3 contrasts shown in Figure 5B are tabulated.
